# Reversal of Endothelial Cell Anergy by T Cell-Engaging Bispecific Antibodies

**DOI:** 10.3390/cancers16244251

**Published:** 2024-12-20

**Authors:** Márcia Gonçalves, Karsten M. Warwas, Marten Meyer, Reinhard Schwartz-Albiez, Nadja Bulbuc, Inka Zörnig, Dirk Jäger, Frank Momburg

**Affiliations:** 1 Clinical Cooperation Unit Applied Tumor Immunity, German Cancer Research Center (DKFZ), 69120 Heidelberg, Germany; m.goncalves@dkfz-heidelberg.de (M.G.);; 2 Antigen Presentation and T/NK Cell Activation Group, German Cancer Research Center (DKFZ), 69120 Heidelberg, Germany; 3 Department of Medical Oncology, National Center for Tumor Diseases (NCT), University Hospital Heidelberg, 69120 Heidelberg, Germany

**Keywords:** bispecific antibodies, tumor vasculature targeting, T cell activation, transendothelial migration, tumor immunotherapy

## Abstract

**Objectives:** Reduced expression of adhesion molecules in tumor vasculature can limit infiltration of effector T cells. To improve T cell adhesion to tumor endothelial cell (EC) antigens and enhance transendothelial migration, we developed bispecific, T-cell engaging antibodies (bsAb) that activate T cells after cross-linking with EC cell surface antigens. **Methods:** Recombinant T-cell stimulatory anti-VEGFR2–anti-CD3 and costimulatory anti-TIE2–anti-CD28 or anti-PD-L1–anti-CD28 bsAb were engineered and expressed. Primary lines of human umbilical vein endothelial cells (HUVEC) that constitutively express VEGFR2 and TIE2 growth factor receptors and PD-L1, but very low levels of adhesion molecules, served as models for anergic tumor EC. **Results:** In cocultures with HUVEC, anti-VEGFR2–anti-CD3 bsAb increased T cell binding and elicited rapid T cell activation. The release of proinflammatory cytokines TNF-α, IFN-γ, and IL-6 was greatly augmented by the addition of anti-TIE2–anti-CD28 or anti-PD-L1–anti-CD28 costimulatory bsAb. Concomitantly, T cell-released cytokines upregulated E-selectin, ICAM1, and VCAM1 adhesion molecules on HUVEC. HUVEC cultured in breast cancer cell-conditioned medium to mimic the influence of tumor-secreted factors were similarly activated by T cell-engaging bsAb. Migration of T cells in transwell assays was significantly increased by anti-VEGFR2–anti-CD3 bsAb. The combination with costimulatory anti-TIE2–anti-CD28 bsAb augmented activation and proliferation of migrated T cells and their cytotoxic capacity against spheroids of the MCF-7 breast cancer cell line seeded in the lower transwell chamber. **Conclusions:** T cells activated by anti-VEGFR2–anti-CD3 and costimulatory EC-targeting bsAb can reverse the energy of quiescent EC in vitro, resulting in improved T cell migration through an EC layer.

## Simple Summary

Rich cytotoxic T-cell infiltration in cancerous tissues is associated with better clinical prognosis. A major obstacle to tumor T cell infiltration can be the scarcity of adhesion molecules expressed by the growing tumor neo-vasculature that limits the binding and extravasation of effector lymphocytes. In the present study, we aimed at reversing this endothelial cell anergy in vitro by using bispecific antibodies that redirect T cells to endothelial cell growth factor receptors and mediate T cell activation via CD3 and costimulation via CD28 in direct contact with endothelial cells. Antibody-mediated cross-linking of T cells and endothelial cells resulted in a profound upregulation of adhesion molecules due to the secretion of proinflammatory T cell cytokines. T-cell adhesion to previously quiescent endothelial cells, as well as their capacity to migrate through an endothelial cell monolayer and subsequently kill breast cancer cell spheroids, was greatly augmented.

## 1. Introduction

The migration of effector lymphocytes from the bloodstream into the tumor microenvironment is a crucial step for the anti-tumor defense of the immune system [1,2]. There is evidence that the tumor neo-vasculature limits the infiltration of effector T cells into cancerous tissues by a phenomenon termed endothelial cell (EC) energy [3,4]. Tumors with poor T cell representation show active down-regulation of cognate ligands of leukocyte homing receptors on the tumor vasculature, thus limiting the extravasation of potent effector cells [3,4,5,6,7]. The VEGFR2-inhibiting tyrosine-kinase inhibitor sunitinib and the anti-VEGFA antibody bevacizumab have been shown to reverse tumor endothelial cell anergy and stimulate lymphocyte infiltration in human renal cell cancer treated with these drugs in a neoadjuvant fashion [8]. However, proper dosing of anti-angiogenic compounds is critical, and combinatorial treatments have been used in order to increase efficacy and avoid drug resistance [9,10,11,12]. Therefore, it seems desirable to develop strategies leading to focused tumor microvessel activation that simultaneously can increase the transendothelial migration of effector T and NK cells into the tumor tissue. We reasoned that tumor EC activation could be facilitated by focused secretion of pro-inflammatory cytokines resulting from T cell activation after antibody-mediated targeting to endothelial cell surfaces. Since activation of VEGFR2 by VEGF is regarded as the most critical driver of tumor angiogenesis, inhibition of the VEGF/VEGFR2 axis by antibodies or decoy receptors capturing VEGF, antibodies blocking VEGFR2 or tyrosine kinase inhibitors inhibiting downstream signaling of VEGFR2, respectively, have been extensively investigated as anti-angiogenic therapeutic strategies [10,13,14].

HUVEC were used in this in vitro study as a phenotypically homogeneous surrogate for true tumor endothelial cells because HUVEC recapitulate an embryonic vascular program resembling tumor endothelial cells with very low or absent expression of adhesion molecules [3,15,16], and constitutively express VEGFR2. To specifically target growing endothelial cells by bispecific T-cell engaging antibodies, we engineered a single-chain antibody version of the clinically approved anti-VEGFR2 antagonist antibody, ramucirumab [13,17,18,19], and incorporated it in a recombinant bispecific (scFv-Fc-scFv)_2_ antibody together with an anti-CD3ε single-chain antibody that activates T cells [20].

Our previous work showed that full T cell activation by bispecific anti-tumor antigen/anti-CD3ε antibodies strongly benefited from the presence of costimulatory bispecific antibodies engaging CD28 on T cells and targeting a second tumor-associated antigen due to the use of a functionally attenuated anti-CD3ε single-chain variable fragment (scFv) antibody format [20]. With the goal to enhance the efficacy of anti-VEGFR2–anti-CD3 bsAb and, at the same time, improve the selectivity of tumor endothelial cell targeting while reducing off-target effects against VEGFR2-expressing normal EC, we employed a costimulatory bsAb targeting the angiopoietin (ANG) receptor TIE2 that is overexpressed in tumor neovasculature and is involved in vascular remodeling [21,22]. With an additional costimulatory bsAb, we targeted the endothelial cell antigen PD-L1. The immune checkpoint molecule PD-L1 blocks T cell activation and cytolysis by binding to the inhibitory PD-1 receptor and regulates transendothelial migration of T cells [23,24,25].

Here, we report that depending on cross-linking to EC, anti-VEGFR2–anti-CD3 bsAb mediated activation of CD8^+^ and CD4^+^ T cells in a process that was strongly enhanced by costimulatory bsAb recognizing TIE2 or PD-L1, respectively. Concomitantly, expression of EC adhesion molecules was induced, promoting the binding of T cells to EC. Following migration through an EC monolayer, T cells were able to elicit the killing of breast tumor cell organoids. However, required the presence of an additional anti-CD3 bsAb cross-linking T cells with a tumor cell antigen.

## 2. Materials and Methods

### 2.1. Primary Cell Isolation, Cell Lines and Culture Conditions

Human peripheral blood mononuclear cells (PBMC) were freshly isolated from a total of 32 buffy coats from healthy donors by density gradient centrifugation. CD3^+^ T cells were purified by negative selection using the pan-T cell isolation kit (Miltenyi Biotec, Bergisch Gladbach, Germany) according to the manufacturer’s instructions. Cell purity was routinely > 95%. Purified CD3^+^ T cells were usually used directly after isolation. Otherwise, they were cultured in RPMI-1640 medium (Merck/Sigma-Aldrich, Darmstadt, Germany) supplemented with 10% heat-inactivated fetal bovine serum (iFBS, Merck Biochrom, Berlin, Germany), 1% penicillin/streptomycin (Sigma-Aldrich) and 2 mM GlutaMAX^TM^ solution (Thermo Fisher/Gibco, Waltham, MA, USA) in the presence of 20 U/mL of IL-2 (Miltenyi Biotec), at 37 °C and 5% CO_2_. The human breast adenocarcinoma cell line MCF-7 (ATCC HTB-22) was used as an EpCAM-expressing cell line. Cells were cultured in a complete RPMI-1640 medium and incubated at 37 °C in a humidified 5% CO_2_ atmosphere. To obtain an MCF-7-conditioned medium, adherent MCF-7 cells were seeded, diluted, and grown to confluency within 3 days. Two days after confluency, the medium was harvested and centrifuged cell-free. Primary human umbilical vein endothelial cells from pooled donors (HUVEC; C-12203, Promocell, Heidelberg, Germany) were cultured in endothelial cell growth medium 2 (ECGM2, Promocell), supplemented with 10% heat-inactivated fetal bovine serum (iFBS), and 1% penicillin/streptomycin at 37 °C in a 5% CO_2_ atmosphere. 4 different batches of HUVEC were used throughout this study. The cells were split when confluency was 80–90% and used until passage 5. HBMEC-60 cells [26] were a kind gift of Prof. C.E. van der Schoot, University of Amsterdam, Amsterdam, the Netherlands. HBMEC-60 cells were grown in a supplemented ECGM2 medium. Freestyle^TM^ CHO-S cells (CHO-S, Gibco) were cultured routinely in PowerCHO-2 CD medium (Lonza, Basel, Switzerland), supplemented with 0.5× Antibiotic Antimycotic solution (Anti-Anti, Sigma-Aldrich) and 8 mM GlutaMAX^TM^ in 500 mL round glass bottles at 37 °C, 100 rpm with a 50 mm shaking diameter in a humidified 8% CO_2_ atmosphere. Human recombinant IL1-β and TNF-α used for EC stimulation were obtained from BioLegend (San Diego, CA, USA).

### 2.2. Cloning of bsAb

The binding moieties of tetravalent bsAb are V_H_–(Gly_4_Ser)_3_-V_L_ scFv derived from monoclonal antibodies, anti(α)-VEGFR2 (Ramucirumab/IMC-1121B, DB05578, US10196447B2); αTIE2 (12H8, US20026376653, US20130209492); αPD-L1 (Avelumab/MSB0010718C; DB11945, PDB:5GRJ_H, PDB:5GRJ_L), αCD3ε (Muromomab/OKT3; DB00075, PDB:1SY6_H, PDB:1SY6_L); αCD28 (9.3; V_H_ GenBank: CAD30987.1, V_L_ GenBank: CAD30986.1). cDNAs coding for the mentioned scFv with endothelial cell specificity were cloned 3′ of an hIg-κ ER leader sequence followed by a glycine-serine-rich linker [“GSL”, GNS(G_4_S)_3_AS] and the hinge-CH2-CH3 domains of hIgG1 (E216–K447) harboring the mutations C220S, E233P, L234A, L235A, DG236, N297Q, K322A, A327G, P329A, A330S, P331S to abolish Fc receptor and complement binding [20]. A StrepTag-II sequence [DPG**WSHPQFEK**SR] flanked by restriction sites BamHI and XbaI was inserted at the 3′ end of the hIgG-Fc sequence. The C-terminal scFv OKT3 and 9.3 sequences were cloned 3′ of the StrepTag-II sequence preceded by a (Gly)_4_ linker. The cDNA sequences coding for mature human IL-1β (GenPept: P01584; A26-S269) and secretory TNF-α (GenPept: P01375; V65-L233) were likewise cloned 3′ of the StrepTag-II sequence of a fusion protein of αVEGFR2 scFv with mouse IgG2a-Fc[C224S/N297Q]-StrepTag-II. The resulting bsAb and bifunctional constructs assemble into covalently linked homodimers due to two intermolecular disulfide bonds in the hIgG1 and mIgG2a hinge regions. All constructs that are listed in Table 1 were cloned between NheI and NotI sites of the expression vector pcDNA3.1(–) (Thermo Fisher/Invitrogen, Waltham, MA, USA).

### 2.3. Production and Purification of bsAb

Transient gene expression (TGE) for protein production based on the CHO-S/ProCHO4/PEI system was performed and optimized as described previously [27,28,29,30]. CHO-S cells were resuspended at 3 × 10^6^ cells/mL in ProCHO4 medium (Lonza) supplemented with 4 mM GlutaMAX followed by sequential addition of 2.5 µg/1 × 10^6^ cells 25 kDa-linear polyethyleneimine (Polysciences, Warrington, PA, USA) and plasmid DNA (maximum of 0.625 µg/1 × 10^6^ cells) directly to the cell suspension [27]. After 6 days in culture at 32 °C, 5% CO_2_, and rotation at 130 rpm, the supernatants of the transfected cultures were harvested and purified using the Strep-Tactin system (IBA Lifesciences, Göttingen, Germany) according to the manufacturer’s instructions. The harvested supernatant was applied to a Strep-Tactin column using a peristaltic pump and washed with PBS (Sigma-Aldrich). Elution of purified proteins was performed by the addition of PBS, supplemented with 5 mM desthiobiotin (IBA Lifesciences). Eluted proteins were dialyzed against PBS, and purity was verified by reducing and non-reducing 10% SDS-PAGE before functional testing. The binding of purified BiMAb was validated on target positive cells (HUVEC and CD3^+^ T cells). Purified proteins were stored at 2–8 °C.

### 2.4. In Vitro Biological Activity of Bispecific Antibodies

To test the biological activity of purified constructs, human-purified CD3^+^ T cells (see Section 2.1) were cocultured with endothelial cells. HUVEC were seeded at a density of 5 × 10^4^ cells per well into a 24-well cell culture plate and cultured in ECGM2 at 37 °C and 5% CO_2_. After 24 h and the formation of a confluent monolayer, HUVEC were treated with the respective bispecific antibodies, and 0.5 × 10^6^ CD3^+^ T cells were added on top. After 4 h or 24 h coculture, non-adherent cells were aspirated, and the remaining cells were carefully washed twice with DPBS. Bound T cells and ECs were detached with enzyme-free PBS-based cell dissociation buffer (Merck/Millipore, Burlington, MA, USA) and washed with DPBS. The number of viable bound T cells was determined by flow cytometry using precision counting beads (BioLegend) according to the respective manufacturer’s instructions using the formulas shown below. The activation status of T cells and HUVEC was analyzed by flow cytometry as described below (Section 2.5).

$$Absolute\;bound\;cells\;count=\left(\frac{Cell\;count\times Precision\;count\;beads\;volume}{Precision\;count\;beads\times Cell\;volume}\times Precision\;count\;beads\;concentration\right)\times Sample\;volume$$

$$Percentage\;of\;bound\;cells=\frac{Absolute\;bound\;cells\;count}{Input\;cells\;count}\times 100$$

### 2.5. Flow Cytometry

HUVEC and purified CD3^+^ T cells were stained with αEC-hIgG-Fc–αCD3/αCD28 BiMAb using 5 μg/mL in FACS buffer (Dulbecco’s PBS (Sigma-Aldrich), 1% bovine serum albumin (PAA Laboratories, Freiburg, Germany), 2 mM EDTA (Sigma-Aldrich) and 0.05% NaN_3_ (Sigma-Aldrich) followed by goat anti-human Ig-PE (Biozol/Dianova, Hamburg, Germany) to evaluate bsAb binding. T cells and HUVEC from coculture and migration assays were harvested to measure activation marker expression or to quantify the binding of T cells to HUVEC by flow cytometry after 4 or 24 h of coculture. Briefly, cells were transferred into 96-well V-bottom plates and washed once with PBS. Dead cells were stained with the Zombie Aqua Fixable Viability Kit (BioLegend). According to the manufacturer’s protocol. Next, Fc receptors were blocked with Human TruStain FcX (BioLegend) and incubated in FACS buffer containing fluorescently labeled monoclonal antibodies for 25 min at 4 °C protected from light. HUVEC were stained with αCD105-AF488 (43A3), αCD62E/E-selectin-PE (HAE-1f), αCD106/VCAM1-APC (STA), αCD54/ICAM1-PE/Cy7 (HA58), αCD202B/TIE2-PE (33.1(Ab33)), αCD309/VEGFR2-APC (7D4-6) and isotype control antibodies mIgG2b-PE (MPC-11), mIgG1-APC/PE (MOPC-21), mIgG2a-APC (MOPC-173). T cells were stained with αCD3-APC-Cy7 (HIT3a), αCD4-A488 (RPA-T4), αCD4-PE (RPA-T4), αCD8-PB (SK1), αCD8-APC (RPA-T8), αCD14-BV510 (M5E2), αCD14-PerCP-Cy5.5 (HCD14), αCD19-BV510 (HIB19), αCD19-A647 (HIB19), αCD355(NKp46)-PE-Cy7 (9E2), αCD25-A647 (BC96), αCD56-BV510 (HCD56), αCD134/OX40-PE-Cy7 (ACT35), and αCD137(4-1BB)-PE (4B4-1). Cells were washed twice prior to flow cytometric measurement. For T cell quantification, precision counting beads were used. FlowJo software (v10.6.2, TreeStar Inc., Ashland, OR, USA) was used for analysis. A minimum of 1 × 10^4^ living CD3^+^ T cells or HUVEC were acquired for each sample. All fluorochome-conjugated monoclonal antibodies were purchased from BioLegend. In some experiments, purified T cells were labeled with 1 µM CellTrace Violet (CTV, Thermo Fisher Scientific) following the manufacturer’s instruction to assess T cell proliferation or to track T cells. CTV-labeled T cells were cocultured with target cells and bsAb for a period of three days, and the proliferation of CD4^+^ and CD8^+^ T cells, based on CTV dilution due to cell division, was examined using flow cytometry.

### 2.6. Enzyme-Linked Immunosorbent Assay (ELISA)

Cytokine levels (TNF-α, INF-γ, IL-6) were determined by sandwich ELISA using a kit from BioLegend. The capture antibody was resuspended in coating buffer and added to the 96-well ELISA plate overnight at 4 °C. After washing; the wells were blocked with blocking buffer for 45 min at 37 °C. After three washing steps, samples were diluted with sample buffer and transferred to each well in duplicates. Depending on the type of cytokine measured, the sample dilution was adjusted (TNF-α 1:5 dilution, INF-γ and IL-6 1:10 dilution). Human cytokine standards were diluted 1:2 (8 dilutions in duplicate) in sample buffer to make the standard curve. After 90 min of incubation at RT and three washing steps, the detection antibody diluted in sample buffer was added to each well for 60 min at RT. After washing 3 times, streptavidin-HRP was added and incubated at RT for 20 min. After 3× washing, substrate solution was added for 10 min at RT, and the reaction was stopped by the addition of a stop solution. Absorbance at 450 nm/540 nm was measured using an ELISA plate reader (Multiskan EX, Thermo Scientific, Waltham, MA, USA), and the final concentrations were determined using the standards (all from BioLegend).

### 2.7. Transendothelial Migration (TEM) Assay

#### 2.7.1. Two-Layer Model

Targeting of T cells to ECs via our bifunctional protein constructs was investigated by a transwell culture system (24-well plate). HUVEC (2.5 × 10^4^ cells) were seeded in fibronectin-coated (15 µg/cm^2^) (0.1% solution, Sigma-Aldrich) cell inserts (5 µm pore size, PET membrane, Merck/Millipore). After 48 h, monolayer confluency was confirmed by FITC-dextran (3–5 kDa; Sigma-Aldrich) permeability assay. Prior to the transmigration assay, the permeability of endothelial cell layers was tested by applying 100 µg/mL FITC-dextran solution (diluted in ECGM2) to the upper well. After 1 h at 37 °C, medium from the lower well was collected, and the dextran concentration was determined in duplicates measuring excitation at 485 nm and emission at 538 nm using a Fluoroskan Ascent FL spectrophotometer (Thermo Scientific). Dextran diffusion was quantified using a standard curve. After washing twice with PBS, bifunctional constructs were added to the upper well, followed by 0.5 × 10^6^ T cells/well. Transmigration took place for 24 h at 37 °C and 5% CO_2_ in ECGM2 under static conditions. The number of viable transmigrated T cells in the lower chamber was determined by flow cytometry using precision counting beads according to the respective manufacturer’s instructions. T cell and HUVEC activation status was studied by FACS as described above (Section 2.5).

$$Migrated\;cell\;count=\left(\frac{Cell\;count\times Precision\;count\;beads\;volume}{Precision\;count\;beads\times Cell\;volume}\times Precision\;count\;beads\;concentration\right)\times Sample\;volume$$

$$Percentage\;of\;migration=\frac{Migrated\;cell\;count}{Input\;cell\;count}\times 100$$

#### 2.7.2. Three-Layer Model and Tumor Spheroid Generation

MCF-7 were grown in 100 µL DMEM supplemented with B-27 supplement and 1% Geltrex^TM^ basement membrane matrix (Gibco). 5 × 10^4^ cells/well were seeded in low-adhesion 96-well round bottom plates (Thermo Fisher), centrifuged at 100× *g* for 5 min and maintained at 37 °C, 5% CO_2_ in a humidified incubator. Spheroids formed overnight after seeding and were incubated for 2 days before functional experiments. Five spheroids were used in each lower well of a low-adhesion 24 well-plate (Thermo Fisher) and kept in 600 µL DMEM medium supplemented with B-27 supplement (no FBS) with or without αEpCAM-reactive bsAb at 10 nM final concentration. This bsAb can crosslink migrated T cells via αCD3ε and MCF-7 via EpCAM binding [20], leading to an additional stimulation of T cells and tumor cell targeting. Transwell inserts, prepared as described above (Section 2.7.1), were transferred into the spheroid-containing plate, and T cells, as well as anti-EC bsAb, were added to the insert in 200 µL EC medium supplemented with 1% FBS. Transmigration took place for 24 h at 37 °C and 5% CO_2_ under static conditions. Transwell inserts were then removed, and migrated T cells were left in cocultures with the spheroids embedded in Geltrex for another 48 h. After a total incubation time of 72 h, cells were collected to evaluate T-cell activation and proliferation.

### 2.8. In Vitro Cytotoxicity Assay

The cytotoxicity capacity of migrated T cells (Section 2.7.2) or CD3^+^ T cells from 24 h cocultures (see Section 2.4) was analyzed using the CyQUANT LDH Cytotoxicity Assay kit (Thermo Fisher), according to the manufacturer’s instructions. Briefly, the supernatants from T cell/HUVEC (24 h) or migrated T cell/MCF-7 (72 h) cocultures were transferred to a 96-well plate, and substrate mix solution from the LDH kit was added on top for 30 min in the dark at RT. To stop the reaction, a stop solution was added, and the plate absorbance was measured with the Multiskan EX reader at 492 nm/620 nm. Maximal lysis of target cells was achieved by incubation of target cells with lysis buffer. Spontaneous LDH release refers to target and effector cells without bsAb. Background medium absorbance was also subtracted before applying the following equation:

$$\%\;Cytotoxicity=\frac{Sample\;release-effector\;cells\;spontaneous\;release-target\;cells\;spontaneous\;release}{Target\;cell\;maximum\;release-target\;cells\;spontaneous\;release}\times 100$$

### 2.9. Statistical Analysis

Statistical analyses were performed using GraphPad Prism 7. All the data are expressed as mean ± SEM. Experiments containing more than two experimental groups were analyzed using a one-way or two-way analysis of variance (ANOVA) with Tukey’s multiple comparison test or Dunnett’s follow-up test where appropriate. The number of donors and experiments, as well as the statistical analysis, is stated in the respective figure legends with *p* values < 0.05 considered statistically significant (ns, *p* ˃ 0.05; *, *p* < 0.05; **, *p* < 0.01; ***, *p* < 0.001; ****, *p* < 0.0001).

## 3. Results

### 3.1. Novel Anti-EC–Anti-T Cell Bispecific Antibodies

**Figure 1:**
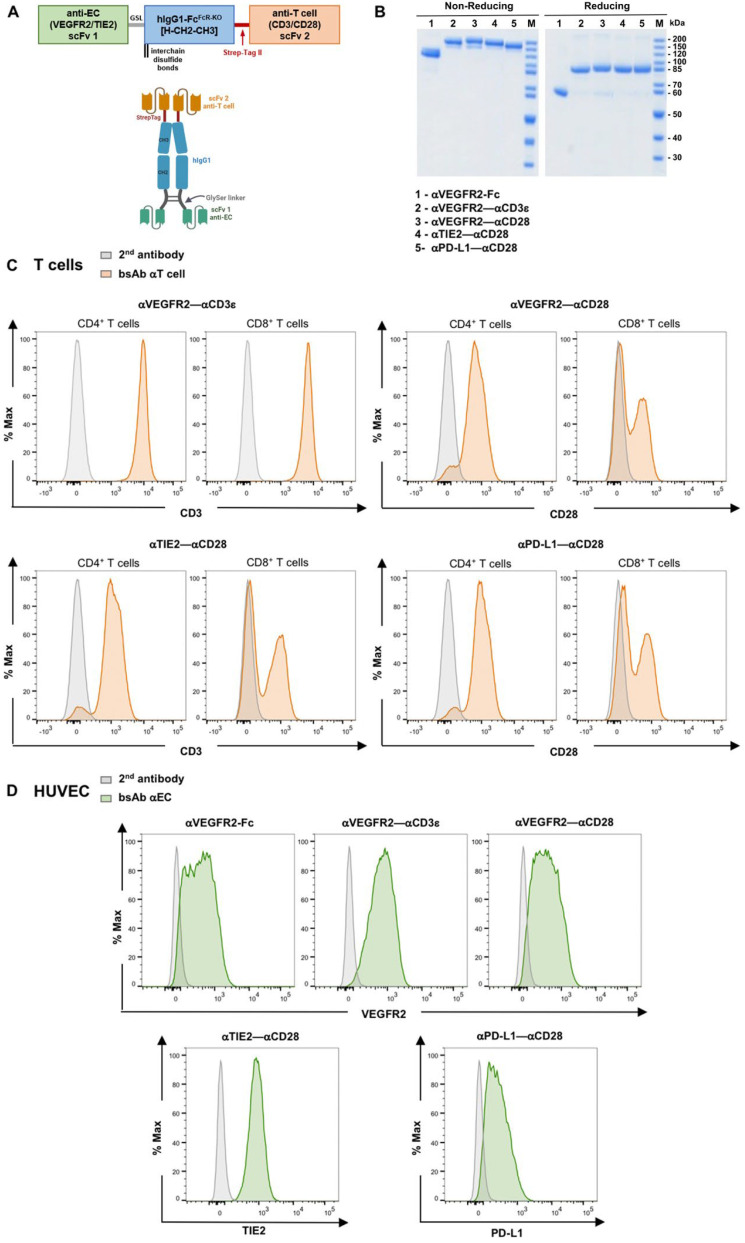
Binding of bsAb to CD3^+^ T cells and to endothelial cells. (**A**) Schematic representation of the tetravalent bispecific bsAb format used in this work. A single chain variable fragment (scFv) antibody 1 specific for tumor endothelium surface receptors is linked to the hinge–CH2–CH3 domains of hIgG1 via a flexible glycine-serine linker (GSL). The Fc domain contains multiple point mutations to abrogate the Fc receptor and complement binding. At the C-terminal end of the CH3 domain, a Strep-tag II is added for immunoaffinity purification, followed by an scFv antibody 2 recognizing either CD3ε or CD28. (**B**) SDS-PAGE analysis (10%) and Coomassie staining of purified bispecific antibodies under non-reducing and reducing conditions. (**C**) Freshly isolated T cells and (**D**) HUVEC were incubated with 5 mg/mL of produced constructs, αVEGFR2–αCD3ε, αTIE2–αCD28, αPD-L1–αCD28, and binding was detected by goat anti-human-IgG1-PE secondary antibody which was also used alone for a negative staining control (grey histograms).

Tumor endothelium is characterized by an abnormal vascular network that can have a strong impact on the efficacy of anti-tumor immune responses due to the lack of properly expressed adhesion molecules responsible for binding and transendothelial migration of effector immune cells. To redirect T cells to tumor endothelial cells and potentially overcome endothelial cell anergy, a panel of tetravalent bispecific antibodies in the (scFv1-hIgG1-Fc-scFv2)_2_ format were engineered to target T cells to tumor endothelium and to activate them in situ (Figure 1A and Table 1, see Section 2.2). scFv antibodies specific for tumor endothelium growth factor receptors, VEGFR2 or TIE2, and the immune checkpoint molecule PD-L1, respectively, were conjugated to the hinge–CH2–CH3 domains of hIgG1 via a flexible glycine-serine linker. The hIgG1-Fc domain was mutated to abrogate Fc receptor binding. At the C-terminal end of the CH3 domain, a Strep-tag II was added for affinity purification, followed by a second scFv antibody recognizing either CD3ε or CD28. Recombinant proteins were produced by transient gene transfection in CHO-S cells, purified by StrepTactin affinity chromatography, and analyzed by SDS-PAGE (Figure 1B).

To analyze the binding of produced constructs, purified CD3^+^ T cells or HUVEC were incubated with purified αVEGFR2–αCD3ε, αVEGFR2–αCD28, αTIE2–αCD28 or αPD-L1–αCD28 bsAbs and stained with a secondary antibody. The αVEGFR2–αCD3ε bsAb bound homogenously to CD4^+^ and CD8^+^ T cells (Figure 1C). Using αCD28 bsAbs, larger and smaller CD28^−^ subpopulations were observed in peripheral blood CD8^+^ and CD4^+^ T cells, respectively, consistent with previous reports showing that CD28 expression can be variable and donor-dependent [20,31]. We also assessed the binding of αVEGFR2–αCD3ε, αVEGFR2–αCD28, αTIE2–αCD28, αPD-L1–αCD28 bsAbs as well as an αVEGFR2-Fc control protein to HUVEC. Positive HUVEC staining confirmed the constitutive cell surface expression of VEGFR2, TIE2, and PD-L1 (Figure 1D).

### 3.2. T Cell Activation by bsAb-Mediated Targeting to Endothelial Cells

**Figure 2:**
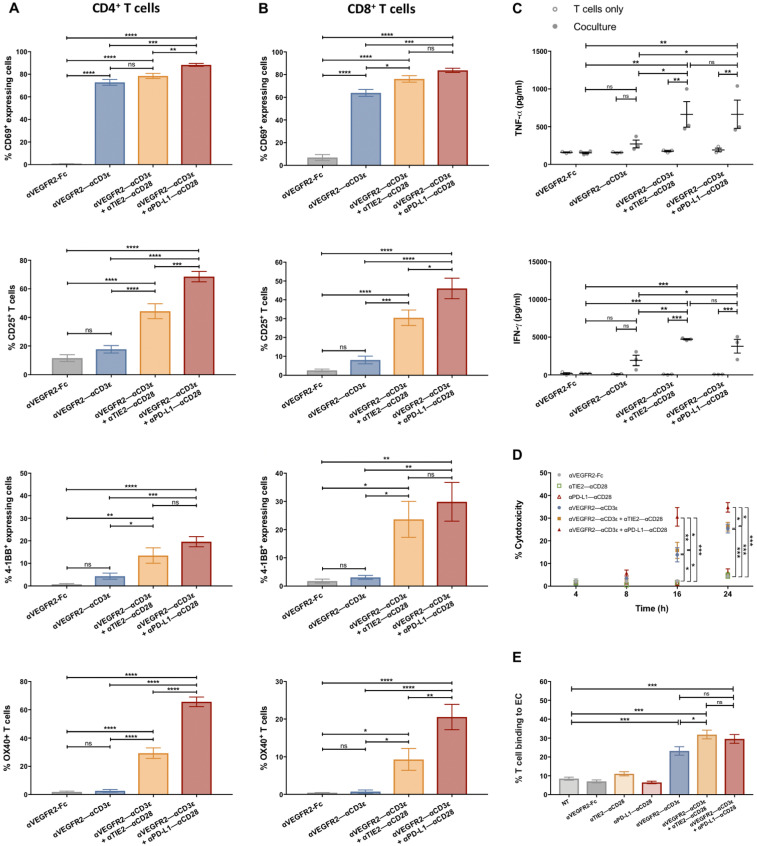
T cell activation induced by αEC bsAbs. Freshly isolated T cells were cocultured with HUVEC in the presence of different bsAb, αVEGFR2–αCD3ε (1 nM) alone or in combination with αTIE2–αCD28 (1 nM) or αPD-L1–αCD28 (1 nM) for 24 h. After three washing steps, firmly EC-bound T cells were collected, and the induction of activation surface markers (CD69, CD25, 4-1BB, and OX40) was studied on (**A**) CD4^+^ and (**B**) CD8^+^ T cells using flow cytometry. Data are presented as means of percentages of T cells expressing the indicated activation markers ± SEM from 6 independent experiments. (**C**) Supernatants from the 24 h coculture were collected, and TNF-α and IFN-γ secretion was quantified by sandwich ELISA. Data are presented as mean values ± SEM from 3 independent experiments. (**D**) T-cell cytotoxic effects in HUVEC cocultures were studied at the indicated time points (4 h–24 h) by quantification of LDH released to cell culture supernatants. Data is presented as mean ± SEM from 3 independent experiments. (**E**) Firm T cell binding to EC was quantified using precision counting beads. Data are presented as means of percentages of bound cells from input ± SEM from 3 independent experiments. Statistical analysis by one-way ANOVA test followed by Tukey’s multiple comparison test (**A**,**B**,**D**,**E**) or two-way ANOVA followed by Sidak’s multiple comparison test (**C**); ns, not significant; * *p* < 0.05; ** *p* < 0.01; *** *p* < 0.001, **** *p* < 0.0001.

By flow cytometry, we analyzed bsAb-mediated T cell activation after coculture for 24 h with HUVEC in the presence or absence of bsAb for CD4^+^ T cells (Figure 2A and Supplementary Figure S1A) and CD8^+^ T cells (Figure 2B and Supplementary Figure S1B) using antibodies against the early activation marker CD69 and the late activation molecules CD25, 4-1BB and OX40. First, bsAb was titrated between 0.001 and 10 nM to determine concentrations needed for saturating T cell responses. For CD69 expression, this was achieved at 1 nM of αVEGFR2–αCD3ε bsAb and combinations with either 1 nM αPD-L1–αCD28, αVEGFR2–αCD28 or αTIE2–αCD28 bsAb (Supplementary Figure S1A,B), which were therefore chosen as the standard bsAb concentration in Figure 2 and subsequent experiments. The dose-dependent activation of T cells by the αVEGFR2–αCD3ε bsAb was entirely dependent on the presence of HUVEC in cocultures (Supplementary Figure S1A,B). The αVEGFR2–αCD28 bsAb only had a weak costimulatory effect that could be explained by competition for limited VEGFR2 target antigen expressed by HUVEC. This finding led to the exclusion of this costimulatory bsAb from further experiments.

Of note, neither of the costimulatory bsAbs, αVEGFR2–αCD28, αTIE2–αCD28, or αPD-L1–αCD28 alone did elicit any T cell activation at 10 nM concentration (Supplementary Figure S1). Moreover, T cell coculturing with HUVEC in the presence of a control αEpCAM–αCD3ε bsAb targeting the epithelial antigen EpCAM did not result in T cell activation, clearly demonstrating the need for T–HUVEC crosslinking. When bsAb were added to T cells in the absence of HUVEC in 24 h cultures, a minor degree of target cell-independent T cell activation was observed using the combinations αVEGFR2–αCD3ε/αPD-L1–αCD28 at 1 and 10 nM and αVEGFR2–αCD3ε/αTIE2–αCD28 at 10 nM (Supplementary Figure S1A,B), which could be related to expression of PD-L1 and VEGFR2 on small T cell subsets enabling bsAb-mediated CD3 crosslinking and subsequent costimulation among T cells [20,32,33]. For the late activation markers, CD25, 4-1BB, and OX40 saturating responses were achieved within 24 h only for the combination of αVEGFR2–αCD3ε with αPD-L1–αCD28 at 1 nM (Supplementary Figure S1A,B). While for induction of CD69 on CD4^+^ and CD8^+^ T cells, the αVEGFR2–αCD3ε bsAb alone at 1 nM was sufficient (Figure 2A,B, top panels), costimulation by αPD-L1–αCD28 or αTIE2–αCD28 bsAb was required to achieve significant upregulation of CD25, 4-1BB, and OX40 (Figure 2A,B), confirming that the OKT3 αCD3ε scFv alone led to incomplete T cell activation (20). Regarding OX40 and 4-1BB upregulation, costimulation by the αPD-L1–αCD28 bsAb was stronger than by the αTIE2–αCD28 bsAb, as shown by lower concentrations needed for half-maximal responses (Supplementary Figure S1C). During a 24 h coculture with HUVEC in the presence of stimulatory and costimulatory bsAb, PD-1 expression on previously naïve T cells was not significantly upregulated, nor was PD-L1 expression on HUVEC significantly influenced (Supplementary Figure S2A).

Supernatants from the same HUVEC coculture experiments were analyzed for the presence of TNF-α, IFN-γ, and IL-6 (Figure 2C and Supplementary Figure S2). In accordance with the upregulation of activation markers, induction of TNF-α, IFN-γ, and IL-6 was significantly increased in the presence of the costimulatory αTIE2–αCD28 and αPD-L1–αCD28 bsAb (Figure 2C). In the presence of 1 nM bsAb, costimulatory bsAb alone, culturing of T cells without HUVEC, nor the use of a control αEpCAM–αCD3ε bsAb targeting an epithelial antigen, stimulated cytokine secretion (Figure 2C and Supplementary Figure S2B–D), demonstrating that T cells required crosslinking to HUVEC for activation.

Next, we studied whether T cells were able to kill HUVEC in cocultures in the presence of bsAb. Using an LDH release assay, we detected a small degree of cytotoxicity elicited by the T cell-activating αVEGFR2–αCD3ε bsAb only after 16 h of coculture that slowly increased during the following 8 h (Figure 2D). In line with its more pronounced T cell activation, as shown above, the costimulatory αPD-L1–αCD28 bsAb, but not the αTIE2–αCD28 bsAb, augmented LDH release. As indicated by an increased number of non-adherent CD3^−^ cells that were labeled with ZombieAqua live-dead stain, the release of LDH likely originated from killed and detached HUVEC. In addition to the analysis of the activation status of T cells firmly bound to HUVEC (Figure 2A,B), the adherence of CD3^+^ cells to HUVEC after coculture for 24 h was quantified using precision counting beads. The αVEGFR2–αCD3ε bsAb led to a significantly increased adherence of T cells that was slightly augmented when combined with costimulatory bsAbs (Figure 2E).

### 3.3. Endothelial Cell Activation in Cocultures with T Cells and bsAb

In preliminary experiments, we evaluated the inducibility of adhesion molecules E-selectin, ICAM1, and VCAM1 on primary human umbilical endothelial cells and immortalized HBMEC-60 human brain microvascular endothelial cells in overnight cultures with exogenously added IL1-β, TNF-α or a combination of both. Since VCAM1 was consistently upregulated by HUVEC but inconsistently by HBMEC-60 cells, we decided to conduct T-cell coculture experiments with HUVEC only.

**Figure 3:**
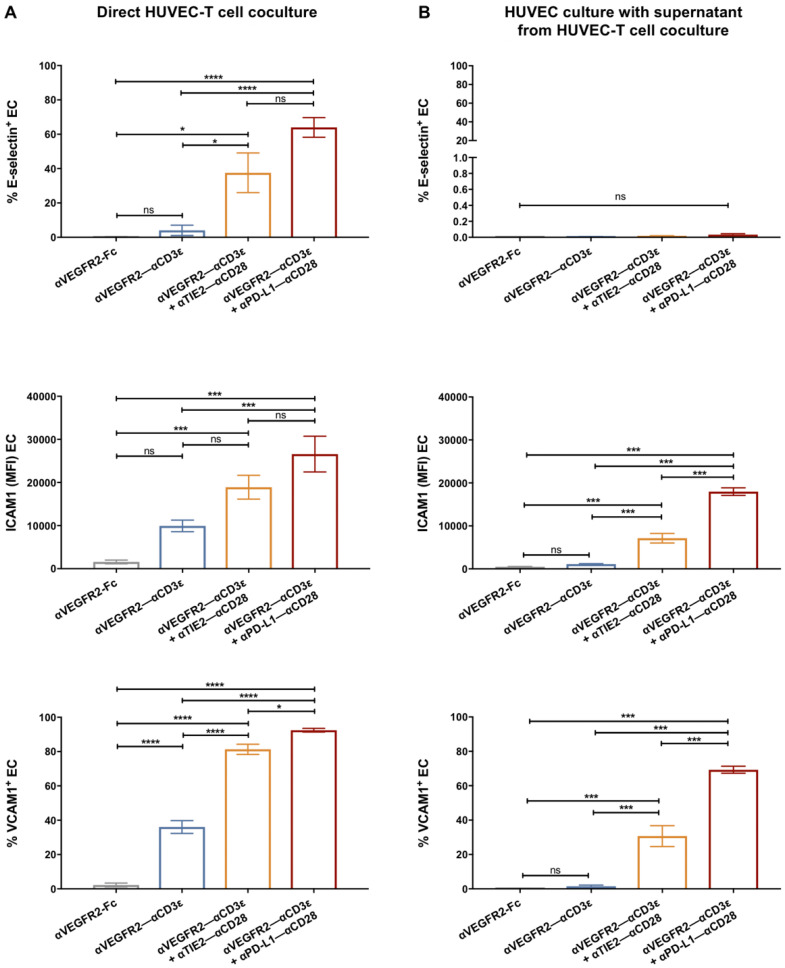
Upregulation of adhesion molecules by HUVEC in the presence of bsAb-activated T cells. (**A**) After 24 h of HUVEC–T cell coculture in the presence of the indicated bsAb, EC was dissociated from plates, and expression of adhesion molecules E-selectin, VCAM1, and ICAM1 was evaluated by flow cytometry. (**B**) Supernatants from HUVEC–T cell cocultures were collected after 24 h and transferred to an HUVEC monolayer. Expression of adhesion molecules E-selectin, VCAM1, and ICAM by HUVEC was evaluated after 24 h by flow cytometry. Data are presented as mean values ± SEM from 3 independent experiments. Statistical analysis by one-way ANOVA followed by Tukey’s multiple comparison test; ns, not significant; * *p* < 0.05; *** *p* < 0.001; **** *p* < 0.0001.

**Figure 4:**
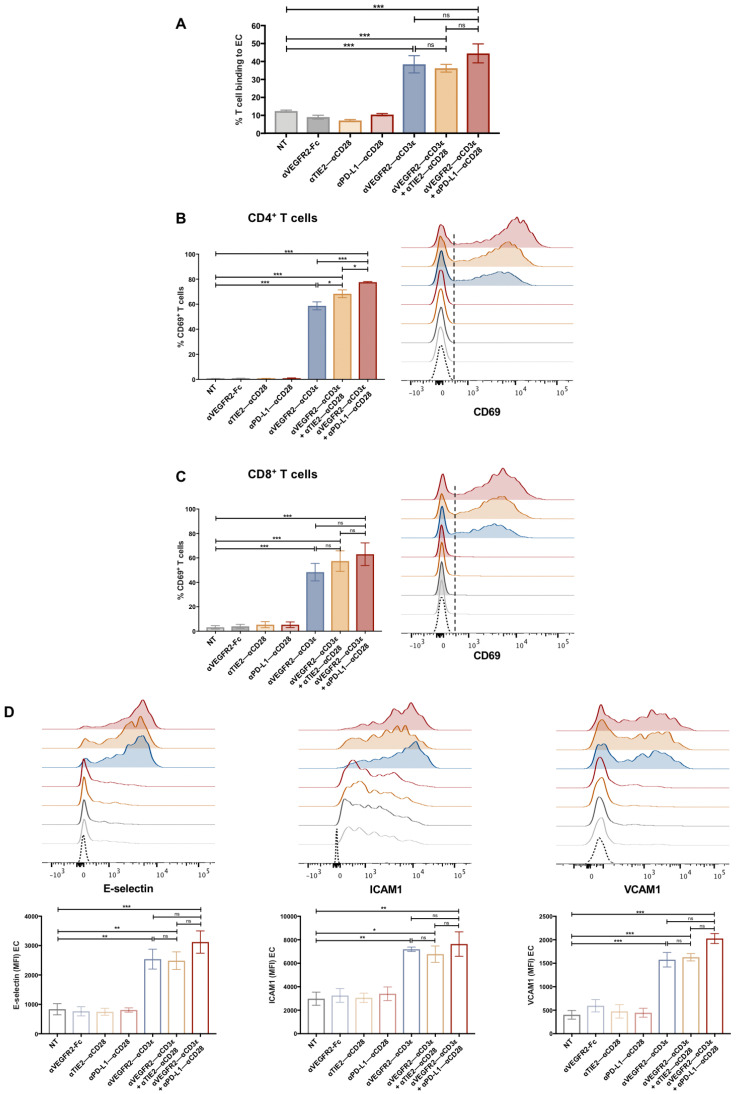
Rapid enhancement of T cell adhesion to HUVEC by αVEGFR2–αCD3ε bsAb. Freshly isolated T cells were cocultured with adherent HUVEC with or without bispecific antibodies αVEGFR2–αCD3ε (1 nM) alone or in combination with αTIE2–αCD28 (1 nM) or αPD-L1–αCD28 (1 nM) for 4 h. After extensive washing, firmly bound T cells were collected by cell dissociation buffer and analyzed by flow cytometry. (**A**) Percentage of firm T cell binding from T cell input number was quantified using precision counting beads. (**B**,**C**) CD69 expression, which was used as a T cell early activation marker, was assessed by flow cytometry for CD4^+^ T cells (**B**) and CD8^+^ T cells (**C**). (**D**) Upregulation of cell surface expression of adhesion molecules E-selectin, VCAM1, and ICAM1 on HUVEC after 4 h of coculture was measured by flow cytometry. Data are presented as mean ± SEM from 3 independent experiments. Statistical analysis was done by one-way ANOVA test followed by Tukey’s multiple comparison test; ns, not significant; * *p* < 0.05; ** *p* < 0.01; *** *p* < 0.001.

Because endothelial cell adhesion molecules are critical for T lymphocyte binding and subsequent extravasation into tumor tissues, we analyzed whether adhesion molecule expression by HUVEC was influenced by coculture with T cells. The cell surface expression of adhesion molecules E-selectin, ICAM1, and VCAM1 on HUVEC cells was studied by flow cytometry after a 24 h coculture with T cells in the presence of bsAb (Figure 3A). In line with the anergic phenotype of unstimulated HUVEC, after coculture with an αVEGFR2-Fc control protein, virtually no expression of E-selectin and VCAM1 was detectable, while ICAM1 showed a low constitutive expression. The αVEGFR2–αCD3ε bsAb induced a significant upregulation of VCAM1, and a tendency to upregulate ICAM1 was noted. After coculture with the costimulatory combination αVEGFR2–αCD3ε/αTIE2–αCD28 and, even more so, with αVEGFR2– αCD3ε/αPD-L1–αCD28, we observed a significant induction of E-selectin, ICAM1, and VCAM1 expression levels. In line with a transient upregulation of E-selectin during EC activation [34], we noted, however, that staining intensities for E-selectin after the 24 h coculture were lower as compared with high staining intensities observed after 4 h of coculture (see Figure 4D), while cell numbers of weakly E-selectin^+^ HUVEC remained significantly increased above background in the presence of costimulatory bsAb (Figure 3A).

Next, we asked whether the upregulation of adhesion molecules could be recapitulated by using supernatants from bsAb-stimulated cocultures containing cytokines resulting from T-cell activation (see Figure 2C). Indeed, supernatants from 24 h HUVEC-T cell cocultures in the presence of αVEGFR2–αCD3ε and αTIE2–αCD28 or αPD-L1–αCD28 induced a significant upregulation of VCAM1 and ICAM1, but not of E-selectin (Figure 3B). Incomplete upregulation of ICAM1 and VCAM1 after HUVEC treatment with supernatants suggested that cytokines had been partially depleted by receptor binding or that additional cell-cell contact was involved. To obtain further insight into the nature of cytokines upregulating HUVEC adhesion molecules E-selectin, ICAM1, VCAM1, and integrin α_v_β_3_, we cultured HUVEC in the presence of recombinant IL1-β, TNF-α or a combination of both for 24 h before analysis by flow cytometry (Supplementary Figure S3). IFN-γ had no inducing effect. E-selectin upregulation required treatment with a combination of TNF-α and IL1-β, the latter being majorly produced by myelomonocytic cells that are virtually absent in the coculture with highly enriched CD3^+^ T cells. ICAM1 expression was significantly stimulated to similar levels by either TNF-α or IL1-β or a combination of both. VCAM1 expression was majorly induced by TNF-α, while integrin α_v_β_3_ expression showed high baseline levels that could not be significantly augmented by cytokines. We also studied the influence of TNF-α and IL1-β on VEGFR2, TIE2, PD-L1, and PD-L2 expression levels on HUVEC cells after 24 h of treatment (Supplementary Figure S3). While TIE2 and PD-L1 were not significantly influenced, we noted upregulation of VEGFR2 and PD-L2 by the combination of TNF-α and IL1-β.

To study if T cell binding to HUVEC was rapidly influenced by EC-reactive bsAbs, CD3^+^ T cells were cocultured with HUVEC for 4 h, i.e., at a time point when EC killing was not observed yet (see Figure 2D). T-cell binding was measured using precision counting beads by flow cytometry after carefully removing non-adherent cells. Rapid T cell binding to HUVEC was significantly induced in the presence of the αVEGFR2–αCD3ε bsAb but not in the presence of the αTIE2–αCD28 nor αPD-L1–αCD28 costimulatory bsAb alone (Figure 4A). T cell binding to HUVEC during the short-term coculture was, however, not increased by costimulatory bsAb in spite of CD4^+^ T cells being slightly more activated by costimulatory bsAb. Enhanced T cell adhesion correlated with a significant T cell activation demonstrated by rapid and strong upregulation of the early activation marker CD69 on CD4^+^ and CD8^+^ T cells (Figure 4B,C). Furthermore, after the 4 h adhesion assay, we also observed a quick and highly significant upregulation of EC adhesion molecules E-selectin, ICAM1, and VCAM1 in line with the known rapid TNFα-mediated induction kinetics of these adhesion molecules [34,35] (Figure 4D). In addition, we analyzed the expression of the ICAM1 ligand, LFA1 (ITGAL/ITGB2; CD11a/CD18), and the VCAM1 ligand, VLA4 (ITGA4/ITGB1; CD49d/CD29) on T cells from untreated and bsAb-treated binding assays by flow cytometry. We detected high expression levels of CD11a, CD18, and CD29 and intermediate expression levels of CD49d with minor differences between HUVEC-bound CD4^+^ and CD8^+^ T cell subsets, which were, however, not significantly altered by stimulatory/costimulatory bsAb during the 4 h adhesion assay (Supplementary Figure S4). The results suggest that T–EC cross-linking via the αTIE2/PD-L1– αCD28 bsAb alone was not sufficient for enhanced adhesion but that rather a bsAb-mediated T cell activation and subsequent rapid induction of adhesion molecules on EC was required. Interestingly, costimulation did not detectably enhance T cell adhesion to HUVEC nor upregulation of adhesion molecules on HUVEC, suggesting that T cell activation mediated by the αVEGFR2–αCD3ε bsAb alone was sufficient and exhaustive in this case.

To exclude that monocytes, B cells, NK cells, and other constituents of PBMC influence the capacity of T cells to adhere to HUVEC in the presence of EC–T targeting bsAb and the subsequent activation of T cells, we repeated the adhesion assay using unseparated PBMC. As shown in Supplementary Figure S5A, adhesion of T cells to HUVEC during 4 and 24 h, respectively, and CD4^+^/CD8^+^ T cell activation as determined by upregulation of CD69, were similar to those shown in Figure 2 and Figure 4 using purified T cells. Monocytes, B cells, and NK cells did not significantly adhere to HUVEC, consistent with the absence of CD3ε and CD28 on these non-T cell types.

Since tumor endothelial cells are highly heterogeneous in vivo and phenotypically vary with the tumor microenvironment they were derived from [16,36,37,38]; we sought to mimic the impact of an in vivo tumor microenvironment by exposing HUVEC to a tumor-conditioned medium. When HUVEC and T cells were cocultured for 24 h in medium conditioned for 5 days by growing/confluent MCF-7 cells, we observed an unimpaired upregulation of endothelial cell adhesion molecules E-selectin, ICAM1 and VCAM-1, which was fully depending of αVEGFR2–αCD3ε ± αTIE2/αPD-L1–αCD28 bsAb (Supplementary Figure S5B).

We next asked whether T cell activation and endothelial cell activation could be separated and still would result in increased T cell adhesion. To this end, we engineered bifunctional constructs that are able to retarget IL1-β or TNF-α, respectively, to previously not activated HUVEC. Recombinant human IL1-β and TNF-α were fused to the C-terminus of αVEGFR2 scFv-mIgG2a-Fc^FcR-null^ molecules (Supplementary Figure S6A) and expressed in CHO-S cells. Purified fusion proteins bound to HUVEC similarly to αVEGFR2 scFv-Fc (Supplementary Figure S6B) and proved to be fully functional compared with rhIL1-β or rhTNF-α as they induced E-selectin, ICAM1, and VCAM1 expression with similar preferences as free cytokines (Supplementary Figure S6C). When naïve T cells were cocultured with HUVEC in the presence of αVEGFR2-Fc-IL1-β, αVEGFR2-Fc-TNF-α, or a combination of both, adhesion to HUVEC was significantly increased (Supplementary Figure S6C). T cells that had been preactivated with the anti-CD3ε mAb OKT3 for 3 days showed a tendency to bind more strongly to control-treated HUVEC than naïve T cells, suggesting upregulation of LFA1. Treatment of HUVEC with αVEGFR2-Fc-IL1-β, αVEGFR2-Fc-TNF-α, or a combination of both led to a significantly increased adhesion also of OKT3-preactivated T cells (Supplementary Figure S6D). We conclude that in our in vitro adhesion assay, endothelial cell activation can precede T cell binding, the latter being independently enhanced by prior T cell activation.

### 3.4. T Cell Transendothelial Migration Is Increased in the Presence of bsAb

**Figure 5:**
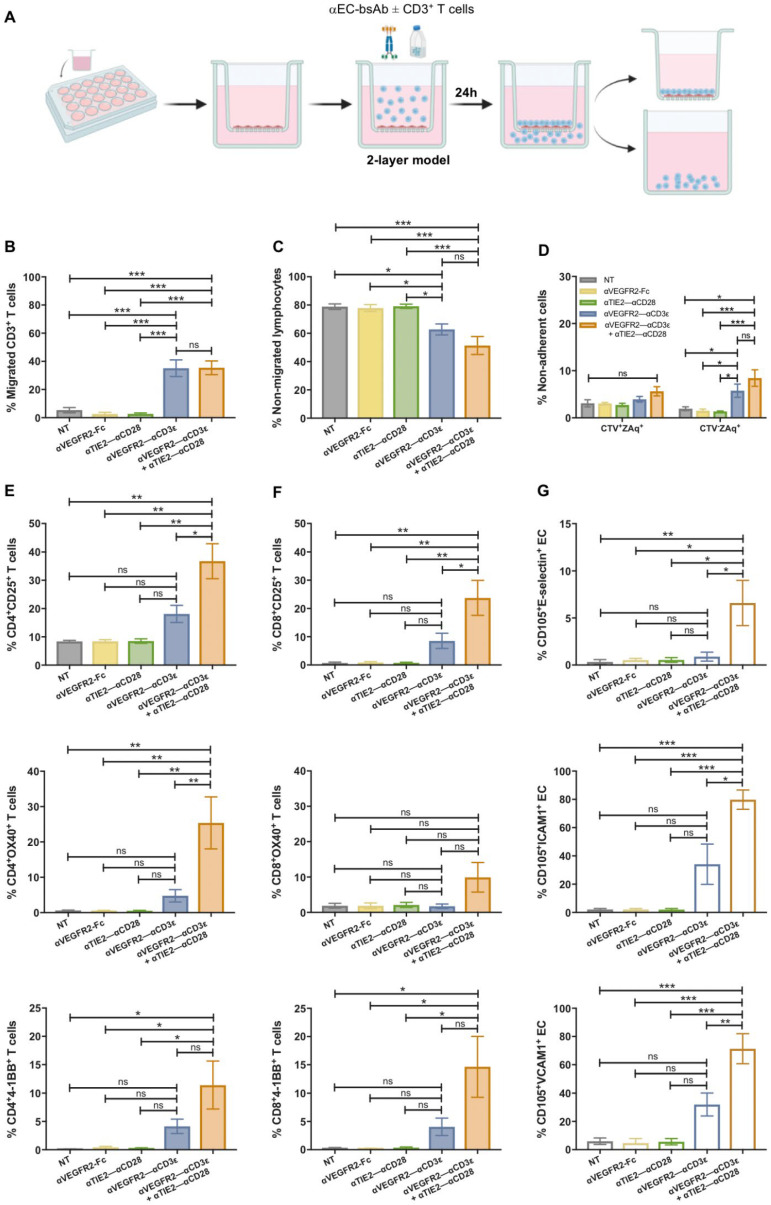
T cell migration and activation are increased by αVEGFR2–αCD3ε and αTIE2–αCD28 bsAb. (**A**) Scheme of the 2-layer transwell model. 2.5 × 10^4^ HUVEC were seeded in transwell inserts coated with 1% human fibronectin and left for 48 h until reaching a tight confluent monolayer. Purified CD3^+^ T cells (0.5 × 10^6^ cells) were added to the insert together with αVEGFR2–Fc (1 nM), αTIE2–αCD28 (1 nM) alone, αVEGFR2–αCD3ε alone (1 nM), αVEGFR2–αCD3ε + αTIE2–αCD28 (1 nM each), or left untreated for control (NT). After 24 h, cells were collected from the lower and upper chambers and migrated CD3^+^ T cells (**B**) (*n* = 6), and non-migrated lymphocytes (**C**) (*n* = 6) were quantified by flow cytometry using counting beads. (**D**) Non-adherent cells from the insert were studied for cell death by uptake of ZombieAqua live/dead stain. CTV-labeled T cells and non-labeled HUVEC were analyzed separately (*n* = 5). The activation of migrated CD3^+^/CD4^+^ (**E**) and CD3^+^/CD8^+^ (**F**) subpopulations was analyzed by measuring expression of CD69, CD25, and 4-1BB by flow cytometry (*n* = 6). A gating scheme is shown in Supplementary Figure S7C. (**G**) Adherent HUVEC harvested from the insert membrane were analyzed for E-selectin (*n* = 6), VCAM1 (*n* = 5), and ICAM1 (*n* = 3) expression by flow cytometry. Data are presented as mean ± SEM from *n* independent experiments done in duplicates. Statistical analysis by one-way ANOVA test (**B**,**D**–**G**) or two-way ANOVA test (**C**) followed by Tukey’s multiple comparison test; ns, not significant; * *p* < 0.05; ** *p* < 0.01; *** *p* < 0.001.

So far, we have shown that T–EC cross-linking is able to induce activation of both T cells and EC. However, the T cell transmigration cascade is a complex process that depends on the expression of suitable adhesion receptors and dynamic interactions between both cell types [39,40]. The influence of αVEGFR2–αCD3ε and αTIE2–αCD28 bispecific antibodies on T cell transendothelial migration was analyzed using a transwell system (Figure 5A). To this end, HUVEC were seeded in fibronectin-coated transwell inserts and grown until confluency. To optimize HUVEC monolayer growth in 48 h, different numbers of seeded HUVEC were tested, and HUVEC monolayer permeability was evaluated by a FITC-dextran (3–5 kDa) permeability test (Supplementary Figure S7A). Results indicated that 2.5 × 10^4^ HUVEC was the optimal seeding number to reach a tight monolayer after 48 h since the percentage of FITC-dextran found in the lower chamber was negligible. After confirmation of the tightness of the HUVEC monolayer, 0.5 × 10^6^ CD3^+^ T cells were added to the upper chamber, together with 1 nM bispecific antibodies for 24 h. No chemokines supporting T-cell migration were added to the lower chamber. A background T cell migration of 5–10% of input T cells was observed in the absence of bsAb condition and in the presence of αVEGFR2–αCD28 bsAb, consistent with 7–12% T cells firmly binding HUVEC in these conditions (see Figure 2E and Figure 4A). This finding could be explained by the fact that there is no flow applied in this model, and T cells will make contact with HUVEC due to gravity and firmly bind via basic ICAM1 expression levels, leading to T cell migration. In the presence of αVEGFR2–αCD3ε, T cell migration increased significantly to approximately 35% of input T cells, while costimulation did not have an additive effect on migration (Figure 5B). Enhanced migration was confirmed by the lower amount of non-migrated lymphocytes found in the transwell insert after bsAbs stimulation compared to the control conditions (Figure 5C). To study the cytotoxic effects of activated CD3^+^ T cells against HUVEC, non-adherent cells from the insert were collected and stained with Zombie Aqua live-dead dye (Figure 5D). T cells added to the insert were labeled with Cell Trace Violet (CTV). In the presence of the αVEGFR2–αCD3ε bsAb, we noted an increased number of dead CTV^−^ cells, suggesting that a proportion of HUVEC had been killed, while the viability of T cells was not significantly affected. Since we were able to recover 3–4 × 10^4^ live adherent HUVEC per insert for subsequent stainings, we assume that the integrity of the EC monolayer was not grossly impaired. Similar to the costimulatory αTIE2–αCD28 bsAb, the costimulatory αPD-L1–αCD28 bsAb also did not enhance endothelial transmigration as compared with αVEGFR2–αCD3ε bsAb alone (Supplementary Figure S7B). Due to its stronger induction of cytotoxicity, the αPD-L1–αCD28 bsAb (see Figure 2D) was, however, not included in transwell experiments.

To confirm an activated phenotype of CD4^+^ (Figure 5E) and CD8^+^ T cells (Figure 5F) after migration, the expression of activation markers CD25, 4-1BB, and OX40 was evaluated for T cells recovered from the lower well. Interestingly, a significant upregulation of activation markers on migrated CD4^+^ and CD8^+^ T cells was entirely dependent on the presence of the costimulatory αTIE2–αCD28 bsAb, suggesting that T cell activation by the functionally attenuated αVEGFR2–αCD3ε bsAb was incomplete and only costimulation in contact with HUVEC had elicited full activation of transmigrated T cells. In agreement with the results of 24 h HUVEC-T cell cocultures in cell culture plates (see Figure 3A), upregulation of E-selectin, ICAM1, and VCAM1 during the 24 h transwell coculture was significantly enhanced by costimulation with the αTIE2–αCD28 bsAb as shown by the flow cytometric analysis of live CD105^+^ HUVEC that were harvested from the insert membrane using enzyme-free cell dissociation buffer (Figure 5G). We also analyzed expression levels of LFA1 and VLA4 on transmigrated T cells after the 24 h transwell coculture. Like in the 4 h coculture (Supplementary Figure S4), we did not detect any upregulation of LFA1 and VLA4 integrins by stimulatory/costimulatory bsAb.

### 3.5. Tumor Cell Cytotoxicity of Transmigrated T Cells After (Co)stimulation on Endothelial Cells

**Figure 6:**
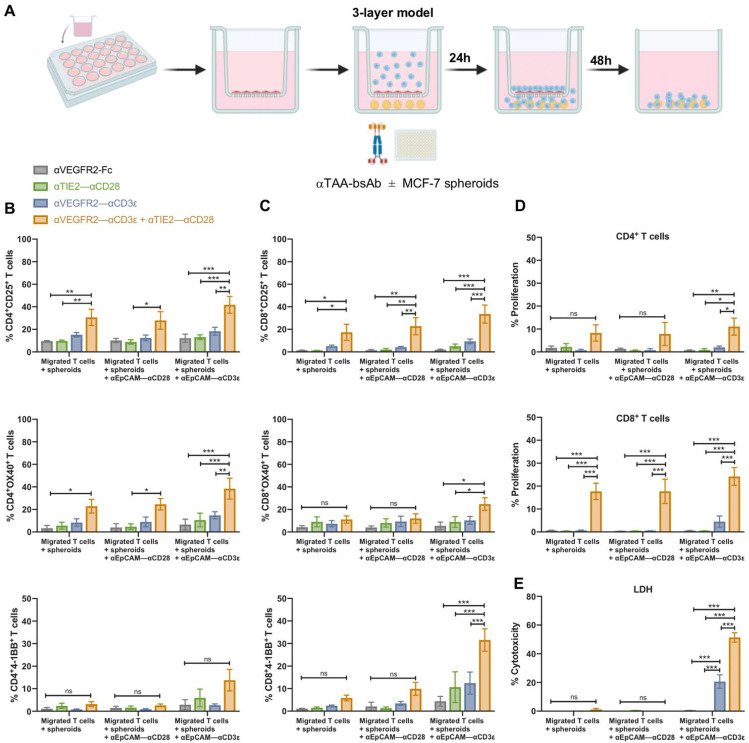
Migrated T cells kill MCF-7 tumor spheroids in the presence of αTAA bsAb. (**A**) Scheme of the 3-layer transwell model. Transwell inserts were coated with 1% human fibronectin, and 2.5 × 10^4^ HUVEC were seeded and left for 48 h until reaching a tight confluent monolayer. Purified CD3^+^ T cells (0.5 × 10^6^ cells) were added to the insert together with αVEGFR2–Fc (1 nM) for control, αTIE2–αCD28 (1 nM), αVEGFR2–αCD3ε (1 nM), or αVEGFR2–αCD3ε + αTIE2–αCD28 (1 nM each). Preformed MCF-7 spheroids were transferred to the lower chamber together with αEpCAM–αCD3 (10 nM) or αEpCAM–αCD28 (10 nM). After 24 h of migration, the inserts were removed, and migrated T cells were kept in culture with the MCF-7 spheroids for an additional 48 h. (**B**) CD4^+^ and (**C**) CD8^+^ T cell activation was analyzed by cell surface staining of migrated cells for CD69, CD25, 4-1BB, and OX40 (*n* = 5). (**D**) CD4^+^ and CD8^+^ T cell proliferation was evaluated by determining intracellular CTV dilution by flow cytometry (*n* = 4). (**E**) Cytotoxicity was assessed by measuring LDH release into the coculture supernatant after 72 h (*n* = 3). Data are presented as mean values ± SEM from 3–6 independent experiments. Statistical analysis by two-way ANOVA test followed by Tukey’s multiple comparison test; ns, not significant; * *p* < 0.05; ** *p* < 0.01; *** *p* < 0.001.

To study the activation, proliferation, and cytotoxic capacity of transmigrated T cells in dependence on prior bsAb-mediated stimulation/costimulation at an endothelial monolayer, we investigated a model system in which MCF-7 breast cancer spheroids were seeded in GeltrexTM basement membrane matrix in the lower well of a transwell chamber. Purified CD3^+^, CTV-labeled T cells were allowed to migrate through an endothelial monolayer in the presence or absence of EC-targeting bsAb for 24 h, before the transwell insert was removed, and the T cells were cultured for an additional 48 h in the presence or absence of an EpCAM-targeting bsAb (Figure 6A). The epithelial antigen EpCAM is strongly expressed in MCF-7 cells [20]. However, MCF-7 cells do not express VEGFR2 nor TIE2 (Supplementary Figure S7D). T cells were harvested and studied by flow cytometry for the expression of late activation markers CD25, OX40, and 4-1BB and for CTV dilution as a marker of proliferation. Furthermore, we measured LDH release to the medium, indicating cytotoxicity. As shown in Figure 6B,C for CD4^+^ and CD8^+^ T cells, respectively, significant CD25 upregulation after 72 h was induced by αVEGFR2–αCD3ε plus αTIE2–αCD28 treatment in the transwell insert, while αVEGFR2–αCD3ε alone was not sufficient. There was a slight additive effect on CD25 upregulation by the αEpCAM–αCD3ε bsAb, suggesting that migrating T cells received an additional activating signal through crosslinking to EpCAM^+^ MCF-7 cells. OX40 tends to be preferentially expressed at higher levels by CD4^+^ T cells and 4-1BB by CD8^+^ T cells, respectively [20]. Accordingly, we observed only a minor upregulation of OX40 on CD8^+^ T cells and 4-1BB on CD4^+^ T cells, respectively. Consistent with the condition for highest CD25 upregulation, OX40 was significantly induced on CD4^+^ T cells and 4-1BB on CD8^+^ T cells using the costimulatory setting in the insert plus αEpCAM–αCD3ε. This condition was also found to significantly induce proliferation of CD4^+^ T cells (Figure 6D). By contrast, the proliferation of CD8^+^ T cells was similarly pronounced if an αEpCAM–αCD28 control bsAb or no αEpCAM bsAb was used, suggesting that (co)stimulation on HUVEC in the transwell insert was sufficient and no further boost by tumor cell crosslinking was required to trigger proliferation of CD8^+^ T cells. LDH release indicating the kill of tumor cells was strictly dependent on the presence of the bsAb αEpCAM–αCD3ε in the lower chamber (Figure 6E). While αEpCAM–αCD3ε treatment alone also elicited cytotoxicity towards MCF-7 cells, we noted a highly significant increment if αTIE2–αCD28 bsAb was included during the initial activation and transmigration period for 24 h. As shown above (Figure 5B), the transmigration rate of T cells treated with αVEGFR2–αCD3ε alone as well as αVEGFR2–αCD3ε plus αTIE2–αCD28 was high, and this treatment was associated with HUVEC cytotoxicity (see Figure 2D and Figure 5D). To normalize for an enhanced T cell migration potentially resulting from disruption of the HUVEC monolayer by cytotoxicity, we repeated the experiment using equal numbers of transmigrated T cells for each treatment condition (5 × 10^4^) to kill MCF-7 spheroids in the presence of αEpCAM–αCD3ε bsAb. As shown in Supplementary Figure S7E, αTIE2–αCD28 bsAb-based costimulation in the insert still induced a significant increase in the αEpCAM–αCD3ε dependent killing of MCF-7 targets, albeit at a lower level. Hence, the stimulatory/costimulatory setting in the transwell insert turned out to be critical to achieving the highest levels of subsequent T cell activation, proliferation, and cytotoxicity against cancer cell spheroids.

## 4. Discussion

Abnormalities of tumor endothelial cells, such as reduced expression of adhesion molecules and deregulated angiogenesis, can result in an insufficient extravasation of leukocytes into tumors [41,42,43,44,45,46]. Furthermore, the tumor microenvironment is characterized by an excess of proangiogenic factors supporting tumor progression and reducing the efficacy of cancer therapies such as chemotherapy, radiotherapy, and immunotherapy [47,48]. Therefore, blockade of tumor angiogenesis together with redirection and local activation of T cells could synergize to potentiate tumor infiltration by immune effector cells and facilitate tumor regression.

In this work, bispecific antibodies were used with the intention to redirect T cells to tumor endothelium in order to mediate T cell activation via CD3 engagement in situ and simultaneously deliver anti-angiogenic effects through T cell cytotoxicity and antibody-mediated blockade of VEGFA binding to VEGFR2 [13,49]. While Kopacek et al. previously published bispecific αCD3–αVEGFR2 antibodies in di-scFv as well as diabody format without analyzing functional properties of the antibodies [50], we first report here the use of αVEGFR2–αCD3ε bispecific antibodies to redirect T cells towards growing endothelial cells in combination with costimulatory EC-targeting antibodies, and study activation and endothelial transmigration of T cells. In a similar approach, a T-cell engager (BiTE) antibody that targets endoglin/CD105 and CD3 was previously used to activate T cells and promote cytolysis of endoglin-expressing cells in a xenograft model [51]. There were, however, concerns regarding an increased expression of endoglin on tumor EC relative to normal EC [52]. Furthermore, αVEGFR2-MICA fusion proteins that bind to the activating receptor NKG2D expressed on NK cells were earlier shown to activate TCRαβ CD8^+^ T cells and subsets of TCRγδ cells [53]. Treatment with αVEGFR2-MICA reduced vascular density in a lung cancer model in mice and improved the infiltration and activation of NK and T cells in the tumor microenvironment. Moreover, αCD16–αVEGFR2 single-domain bispecific antibodies have been used to attract CD16^+^ NK cells and myeloid cells towards tumor endothelium [54].

In an alternative approach that has been extensively studied in preclinical models, VEGFR2-reactive CAR T cells have been successfully employed to attack tumor endothelial cells [55,56,57,58,59]. The efficacy of tumor eradication in mice by VEGFR2-targeting CAR T cells could be improved by co-expression of secreted IL-12 [60] or IL-15 in CAR T cells [61], while no overt toxicities against normal tissues were reported that would generally preclude the use of VEGFR2 as a target for CAR T cells. Additional tumor EC antigens that have been targeted by CAR T cells in preclinical studies were α_v_β_3_ integrin [62], PSMA [63] as well as the EIIIB fibronectin splice variant and PD-L1 [64]. To our best knowledge, CAR T cell therapy addressing the tumor neovasculature has, however, not been investigated in humans.

Bispecific antibodies used in this work were produced in the tetravalent format (scFv1-hIgG1-Fc^KO^-scFv2)_2_ with mutations in the Fc domain to avoid Fc receptor engagement and bystander NK or myeloid cell activation, as previously developed in our lab [20,65]. Hence, the activity of our bispecific agents was independent of FcγR binding, which is relevant for this study since endothelial cells can express FcγR receptors [66].

Importantly, T cell activation by αVEGFR2–αCD3ε essentially relied on the presence of VEGFR2^+^ EC in order to mediate cross-linking between T cells and HUVEC, suggesting that this bsAb format is not prone to activate circulating T cells without contact to growing EC. In HUVEC-T cell cocultures, T cell expression of late activation markers CD25, 4-1BB, and OX40 was significantly enhanced by the presence of the costimulatory bsAb αTIE2–αCD28, and even more markedly by the αPD-L1–αCD28 bsAb. The rapid upregulation of the early T cell activation marker CD69 was if at all, only marginally increased by costimulation. Consistent with a dependency on costimulation for full T cell activation, the newly engineered αVEGFR2–αCD3ε bsAb showed a reactivity profile similar to previously reported αCD3ε bsAb with EpCAM, HER2, EGFR, or CEA tumor antigen specificities, the reactivity of which was strongly augmented by costimulatory bsAb [20].

The weak T cell activation by the stimulatory αVEGFR2–αCD3ε bsAb alone prompted us to use the “split antigen” costimulation approach [20], where costimulation is delivered through an independent antigen expressed on EC targets. The combination with a costimulatory αCD28 bsAb that lacks T cell-activating activity has the advantage that such an approach would allow to reduce the effective dose of the stimulatory αVEGFR2– αCD3ε bsAb and increase the selectivity for those tumor endothelial cells overexpressing VEGFR2 and TIE2, or VEGFR2 and PD-L1, respectively. This would represent a safety measure as resting endothelial cells in healthy tissues are expected to express very low quantities of VEGFR2 and TIE2, except for neo-angiogenesis during wound healing. Nevertheless, careful dose titrations of both stimulatory and costimulatory bsAb need to be conducted in appropriate mouse models with humanized EC target antigens to define dosings mediating sufficient on-target anti-tumor effects and tolerable side effects in healthy tissues before our approach would qualify for clinical development.

Treatment-related adverse side effects of the anti-EGFR2 antibody ramucirumab (Cyramza^®^), which is clinically approved for the treatment of advanced gastric cancer [67], non-small cell lung cancer [18], metastasized colorectal cancer [68] and advanced hepatocellular carcinoma [69], were hypertension, proteinurea, and hyponatraemia as signs of kidney disease, in case of which dose reduction of ramucirumab was recommended [70]. As VEGFR2 is strongly expressed by endothelial cells in glomeruli of the kidney (https://www.proteinatlas.org/ENSG00000128052-KDR/tissue/kidney#img, accessed on 25 January 2024) while TIE2 expression is weak (https://www.proteinatlas.org/ENSG00000120156-TEK/tissue/kidney#img, accessed on 25 January 2024) and PD-L1 (CD274) expression is absent (https://www.proteinatlas.org/ENSG00000120217-CD274/tissue/kidney#img, accessed on 25 January 2024), the split antigen costimulation approach could help to alleviate adverse renal side effects by using low αVEGFR2-Fc-αCD3ε bsAb concentrations. Of note, ramucirumab is a humanized IgG1 monoclonal antibody that not only blocks VEGF-A binding to VEGFR2 and subsequent VEGFR2 activation [13,49] but is also expected to elicit NK cell-mediated antibody-dependent cellular cytotoxicity against endothelial cells through activation of NK cells via FcγRIII as well as activation of monocytes/granulocytes expressing FcγRIII and FcγRI.

In control cultures, we observed a minor degree of HUVEC-independent T-cell activation at high concentrations of αVEGFR2–αCD3ε plus αPD-L1–αCD28 (see Supplementary Figure S1). Since VEGFR2 expression has been reported for CD4^+^ memory T cells and FOXP3^+^ regulatory T cells in the peripheral blood of healthy donors [33,71,72], we assume that VEGFR2–CD3ε crosslinking among T cells resulted in a minor degree of activation, which could have been boosted through the αPD-L1–αCD28 bsAb binding to PD-L1 expressed by activated CD8^+^ and CD4^+^ T cells [32,73]. The minor costimulatory effect of the αTIE2–αCD28 bsAb at high concentrations observed in T cell cultures without HUVEC could be related to residual monocytes in incompletely purified T cell preparations since ~20% of peripheral blood monocytes express the TIE2 receptor [74,75].

Of note, the αTIE2–αCD28 and αVEGFR2–αCD28 bsAb used in this work had a strictly costimulatory function as they had no activity as single agents in the absence of TCR complex CD3ε triggering. An αVEGFR2–αCD28 bsAb was also tested as a costimulatory treatment, but T cell activation was not as high as with the other tested costimulatory bsAbs. Since VEGFR2 is not expressed at high levels on HUVEC, competition between both anti-VEGFR2 constructs may have limited the efficacy of αVEGFR2–αCD28 costimulation. T cell activation could also be achieved by targeting TIE2 with two bsAb incorporating αCD3ε scFv or αCD28 scFv, respectively, but since TIE2 is, in addition to EC, expressed by monocytes, adipocytes, glial cells, and spermatocytes we decided to use the anti-TIE2 bsAb as costimulatory agent only. Because the ANG1/ANG2–TIE2 axis regulates vascular permeability and TIE2 antagonism by ANG2 overexpression in tumors has been implicated in promoting metastasis formation [76,77], an agonistic anti-TIE2 antibody should be preferentially used [21,78,79]. The herein-used mouse anti-hTIE2 antibody 12H8 has been shown to be a TIE2-activating antibody that does not block angiopoietin binding [80].

As an additional approach, we studied an αPD-L1–αCD28 bsAb, which blocks PD-L1 engagement on EC by PD-1 expressed by activated T cells and thus has a dual function of costimulatory EC-targeting bsAb and vascular checkpoint inhibitor. The potent costimulatory capacity of αPD-L1–αCD28 bsAb regarding the activation of T cells binding to tumor-associated antigens has been demonstrated before by us and others [20,81]. PD-L1 expressed by endothelial cells appears to be an attractive target for costimulation since PD-L1 engagement suppresses T cell cytokine synthesis [23], and its blockade has been reported to enhance CD8^+^ T cell activation in the presence of endothelium [24]. In contrast to those earlier studies, we detected constitutive expression of PD-L1 on unstimulated HUVEC (see Figure 1D and Supplementary Figure S3). A downregulation of CD8^+^ T cell activation was also reported for PD-L2 [24], which we found to be constitutively expressed on HUVEC and upregulated by proinflammatory cytokines (see Supplementary Figure S3). Interestingly, murine inducible CD4^+^ regulatory T cells and CD4^+^ effector T cells, but not CD8^+^ T cells, have been reported to engage endothelial PD-L1 using PD-1 or CD80 counter-receptors to facilitate lymphatic transendothelial migration [25]. If that finding also holds for human regulatory T cells in contact with tumor endothelium, endothelial PD-L1 blockade by the αPD-L1–αCD28 bsAb might be useful to impede T_reg_ transendothelial migration.

It has been reported that therapy with ramucirumab in patients with advanced gastric cancer increased tumor infiltration with CD8^+^ T cells that had significantly lower levels of PD-1 compared with PBMC [82]. Moreover, CD45RA^−^FOXP3^high^ CD4^+^ effector regulatory T cells (eT_reg_) were reduced in tumor infiltrates, and the VEGF-dependent proliferation of VEGFR2^+^ eT_reg_ could be blocked by ramucirumab [77]. It will be interesting to investigate whether also the αVEGFR2–αCD3ε bsAb used here is able to block the proliferation of VEGFR^+^ natural T_reg_ present in PBMC [33,71,72].

With the objective of overcoming EC anergy [3,5,83], we reasoned to activate T cells in close proximity to HUVEC by means of bispecific antibodies in order to elicit local secretion of proinflammatory cytokines, that in turn would stimulate HUVEC displaying a phenotype of quiescent tumor endothelial cells with only low levels of ICAM1 expression. The secretion of TNF-α and IFN-γ was significantly enhanced by bsAb-mediated T cell activation and costimulation (see Figure 2). As a consequence, in the presence of T cells and VEGFR-targeting bsAb, HUVEC strongly upregulated the essential adhesion molecules ICAM-1 and VCAM-1 in 24 h HUVEC–T cell cocultures. Substantial upregulation of E-selectin, ICAM1, and VCAM1 was detected already after a 4 h coculture, which showed, however, no significant influence of costimulation. This would be in line with a more protracted, enhancing effect of costimulation on T-cell cytokine secretion.

Upon treatment of HUVEC with supernatants harvested from 24 h, HUVEC-T cell cocultures were able to partially recapitulate the upregulation of ICAM1 and VCAM1 but not of E-selectin. It seems possible that E-selectin upregulation, which poorly responded to TNF-α alone and required monocyte-produced IL-1β (see Supplementary Figures S3 and S6C), required additional stimuli resulting from T cell–HUVEC contacts such as the CD40–CD40L interaction [84]. The firm adhesion of T cells to HUVEC was highly pronounced after the 4 h coculture and slightly declined within 24 h (see Figure 4A and Figure 2E), which is in accordance with the previously reported transient upregulation of E-selectin and VCAM1 [34,35].

Tumor EC from breast cancer tissues has been reported to possess distinctive properties that discriminate them from EC adjacent EC in adjacent normal tissue [16]. Furthermore, single-cell mRNA sequencing of human and mouse tumor EC revealed a profound heterogeneity depending on the tumor microenvironment in vivo [36,37,38]. Due to their rather homogenous phenotype and the absence of potential immunosuppressive factors released by malignant cells and adjacent stromal cells in tumor tissues, the use of pooled primary embryonic EC as an in vitro surrogate for anergic tumor EC may appear an over-simplification that, however, served the purpose of studying in vitro, consistently growing EC with very low to absent adhesion molecule expression. To nevertheless mimic the potential effects of factors secreted by carcinoma cells such as TGF-β [85], we analyzed bsAb- and T cell-mediated changes in adhesion molecule expression by conducting HUVEC–T cell cocultures in the presence of medium conditioned by confluent MCF-7 breast cancer cells. While this approach still neglected the potential influence of inhibitory factors secreted by, e.g., cancer-associated fibroblasts or myeloid-derived suppressor cells, the preserved, full-fledged T cell-mediated adhesion molecule upregulation on HUVEC in the presence of tumor cell-conditioned medium is a first hint that EC-targeting bsAb may be able to overcome endothelial cell anergy also in vivo.

Adhesion of T cells to HUVEC was induced by the αVEGFR2–αCD3ε bsAb, however, it was not significantly enhanced by the two costimulatory bsAb. Since expression levels of T cell adhesion molecules LFA1 and VLA4 engaging ICAM1 and VCAM1, respectively, were not influenced by bsAb during the 4 h HUVEC–T cell coculture and were also not upregulated after the 24 h transmigration assay, our findings suggest that upregulation/de novo expression of ICAM1 and VCAM1 by HUVEC was the decisive factor causing enhanced T cell adhesion. Solely used costimulatory αTIE2–αCD28 and αPD-L1–αCD28 had no augmenting effect on T cell adhesion to HUVEC, being consistent with the complete lack of T cell activation using this condition (see Supplementary Figures S1 and S2). This result confirms that the αCD28 scFv format used for this work is devoid of superagonistic activity, which is in contrast to a previous report showing increased human CD4^+^ T cell adhesion to ICAM-1, VCAM-1 and fibronectin after antibody-mediated crosslinking of CD28 [86].

Upregulation of EC adhesion molecules, E-selectin, ICAM1, and VCAM1, could also be achieved by incubating HUVEC with αVEGFR2-Fc-TNF-α/IL-1β fusion proteins (see Supplementary Figure S6), which could become useful for a targeted preconditioning of anergic endothelial cells in vivo. Also, in this setting, the adhesion of naïve and anti-CD3ε-preactivated T cells to EC was significantly enhanced. The finding that EC activation can be temporally separated from T cell activation suggests that an initial bsAb-mediated EC activation through a first wave of T cells could result in a sustained improvement of T cell binding and extravasation at low bsAb plasma concentrations. This appears to be a realistic scenario in vivo following a single bsAb injection, albeit we previously observed that plasma levels of (scFv-Fc-scFv)_2_ bsAb were relatively stable in mice with a half-life of >72 h after i.v. injection [65].

As a corollary of effective T-cell activation by the VEGFR2-crosslinking bsAb, cytotoxicity against HUVEC was observed (see Figure 2D and Figure 5D). After 16 h of coculture in plates, LDH release was detected and slightly increased towards the 24 h point. As transendothelial migration assays had to be conducted for 24 h in order to obtain sufficient cell numbers for subsequent phenotypic and functional analyses, a certain degree of HUVEC killing in the transwell insert was unavoidable (see Figure 5D). While we assume that the integrity of the EC monolayer was not majorly affected due to the fact that we could recover at least the input number of live HUVEC from the insert membranes, we cannot exclude that the number of T cells that migrated through the fibronectin-coated, 10 µm thick membrane was exaggerated due to partial disruption of the monolayer and spontaneous migration through uncovered pores. 5–10% of T cells migrated through a HUVEC monolayer in the presence of a αVEGFR2-Fc control protein. This was probably due to sedimentation and absence of flow, which can result in prolonged T cell-HUVEC contact independent of the presence of bispecific antibodies. αVEGFR2–αCD3ε bsAb treatment was as effective alone as combined with the costimulatory αTIE2–αCD28 bsAb, leading to about 4 times more transmigrated T cells compared to control. The findings that costimulation did not enhance the rate of transmigration and the costimulatory bsAb alone had no effect were reminiscent of the results of the T cell adhesion assays in plates and suggest that tight adhesion to HUVEC was a prerequisite of T cell transendothelial migration in the transwell system, which was sufficiently triggered by the αVEGFR2–αCD3ε bsAb alone. This is in accordance with the prevalent concept of lymphocyte transendothelial migration, including the steps of tethering/rolling, activation, firm adhesion, and diapedesis [39,40,87]. Our finding that the CD28 antibody used here did not support transendothelial migration on its own is in contrast to a previous report showing that agonistic anti-CD28 antibodies enhanced transendothelial migration and integrin clustering in murine and human memory T cells [88]. On the other hand, the αTIE2–αCD28 bsAb-mediated costimulation markedly supported the upregulation of T cell activation markers CD25, OX40, and 4-1BB (see Figure 5 and Figure 6) and was required to induce proliferation of transmigrated CD8^+^ and CD4^+^ T cells in cocultures with MCF-7 spheroids.

A final important feature was to demonstrate the cytotoxic capacity of bsAb-activated migrated T cells. MCF-7 breast cancer cell spheroids, together with αEpCAM–αCD3ε or αEpCAM–αCD28, were included in the lower well during the transmigration process. Here, we combine an in-vitro transwell model for solid tumors to mimic transmigration through endothelium in order for effector T cells to attack tumor cell compounds embedded in the basement membrane matrix. We believe that this is an interesting preclinical model to which additional layers of complexity could be added, such as mixed spheroids consisting of cancer cells and cancer-associated fibroblasts. Interestingly, prior activation by αVEGFR2–αCD3ε ± αTIE2–αCD28 alone was not sufficient to induce spontaneous cytotoxicity against MCF-7 spheroids. This required the presence of a second activating αEpCAM–αCD3ε bsAb targeting an abundantly expressed tumor-associated antigen, whereas the mere crosslinking of EpCAM and CD28 by an αEpCAM–αCD28 bsAb was not sufficient (see Figure 6E). Tumor cell spheroids of up to ca. 0.5 mm diameters are more difficult to kill than monolayers of adherent tumor cells [20] and, therefore, may have required the additional T cell activation and crosslinking by the αEpCAM–αCD3ε bsAb. We observed a highly significant difference between the cytotoxic capacity of T cells that encountered the αVEGFR2–αCD3ε alone as compared with the combined use of αVEGFR2–αCD3ε and αTIE2–αCD28, advocating a combination of stimulatory and costimulatory pretreatment in order to enhance anti-tumor efficacy. Of note, T cells that underwent spontaneous transendothelial migration without prior EC-focused activation were not eliciting cytotoxicity against MCF-7 spheroids in the presence of our αEpCAM-Fc-αCD3ε bsAb in the lower transwell chamber. While we cannot exclude that an αEpCAM scFv–αCD3ε scFv bispecific T cell engager of smaller molecular size would incite cytotoxicity against MCF-7 also in spontaneously transmigrated, non-activated T cells, our results suggest that combinatorial use of tumor-targeting with EC-targeting bsAb might improve so far unsatisfying clinical results with bsAb targeting solid cancers [89,90,91,92].

## 5. Conclusions

Taken together, the reported results demonstrated the capacity of EC- and T cell-engaging bispecific antibodies to elicit a successful T cell activation upon EC cross-linking and to induce targeted cytokine secretion by T cells. Novel recombinant bispecific antibodies in the tetravalent (scFv-Fc-scFv)_2_ format were engineered for this study. VEGFR2, which is overexpressed on growing EC, was successfully used as a target for a CD3-binding T cell-activating bsAb in combination with costimulatory bsAb targeting CD28 on T cells and the EC surface molecules TIE2 or PD-L1, respectively. This split-antigen costimulation approach has the potential to increase the selectivity for tumor endothelial cells, for which resting fetal umbilical vein endothelial cells served as a surrogate in this study.

Endothelial cell anergy, which has been defined by a lack of tumor-related suppression of the expression of E-selectin, ICAM1, and VCAM1 adhesion molecules, was efficiently overcome in HUVEC-T cell cocultures in the presence of EC-targeting bsAb and could be largely attributed to TNF-α secreted by activated T cells. In transwell assays, T-cell migration through a monolayer of HUVEC was enhanced by EC-targeting bsAb. T cells preactivated by EC-targeting bsAb were able to elicit cytotoxicity against breast cancer cell spheroids after transmigration; however, they required an additional bsAb crosslinking a cancer-associated antigen and CD3.

## Figures and Tables

**Table 1:** Bispecific antibodies and bifunctional recombinant proteins used in this work.

αVEGFR2 scFv–hIgG1-Fc^FcR-null^–αhCD3ε scFv
αTIE2 scFv–hIgG1-Fc^FcR-null^–αhCD28 scFv
αPD-L1 scFv–hIgG1-Fc^FcR-null^–αhCD28 scFv
αVEGFR2 scFv–hIgG1-Fc^FcR-null^–ahCD28 scFv
αEpCAM scFv–hIgG1-Fc^FcR-null^–αhCD3ε scFv [20]
αEpCAM scFv–hIgG1-Fc^FcR-null^–αhCD28 scFv [20]
αVEGFR2 scFv–hIgG1-Fc^FcR-null^ (control)
αVEGFR2 scFv–mIgG2a-Fc^FcR-null^–hTNF-α
αVEGFR2 scFv–mIgG2a-Fc^FcR-null^–hIL-1β
αVEGFR2 scFv–mIgG2a-Fc^FcR-null^ (control)

## Data Availability

The datasets generated and analyzed in the current study are available from the corresponding author upon reasonable request.

## Supplementary Materials

The following supporting information can be downloaded at: https://www.mdpi.com/article/10.3390/cancers16244251/s1, Figure S1: Titration of recombinant bispecific antibodies to achieve optimal T cell activation; Figure S2: Quantification of cytokine secretion after HUVEC-T cell coculture in the presence of EC/T cell-reactive bsAb; Figure S3: Endothelial cell expression profiling after cytokine activation; Figure S4: Integrin expression on T cells after bsAb stimulation for 4 h; Figure S5: A, HUVEC adhesion assay conducted with unseparated PBMC; B, Upregulation of endothelial cell adhesion molecules in the presence of tumor-cell conditioned medium; Figure S6: Anti-VEGFR2-Fc-cytokine fusion proteins increase T cell adhesion; Figure S7: A, Optimization of HUVEC monolayer for transwell assay; B, T cell migration is increased by αVEGFR2–αCD3ε treatment alone or together with αTIE2–αCD28/αPD-L1–αCD28 costimulation; C, Gating strategy to assess detached, dead HUVEC in the non-adherent fraction of transwell inserts; D, Analysis of VEGFR2 and TIE2 expression in MCF-7 cells; E, Cytotoxicity against MCF-7 tumor spheroids by normalized numbers of migrated T cells.

## Abbreviations

ANG: angiopoietin; ANOVA, analysis of variance; α-, anti-; bsAb, bispecific antibody(ies); CHO, Chinese hamster ovary cells; CTV, CellTrace^TM^ Violet; EC, endothelial cells; ELISA, enzyme-linked immunosorbent assay; FBS, fetal bovine serum; GSL, glycine-serine-rich linker; HRP, horseradish peroxidase; HUVEC, human umbilical vein endothelial cells; LDH, lactate dehydrogenase; mAb, monoclonal antibody; MFI, median fluorescence intensity; VEGFR2, vascular endothelial growth factor receptor 2; TAA, tumor-associated antigen(s); scFv, single-chain variable fragment; TMB, 3,3′,5,5′-tetramethyl benzidine.
