# Supplementary material for: Reversal of Endothelial Cell Anergy by T Cell-Engaging Bispecific Antibodies
Source: Cancers (Basel). 2024 Dec 20;16(24):4251. doi: 10.3390/cancers16244251 (PMC11674949; doi:10.3390/cancers16244251)
Supplement: Supplementary file 1 [file cancers-16-04251-s001.zip › cancers-3260429-supplementary.pdf]

Supplementary Materials

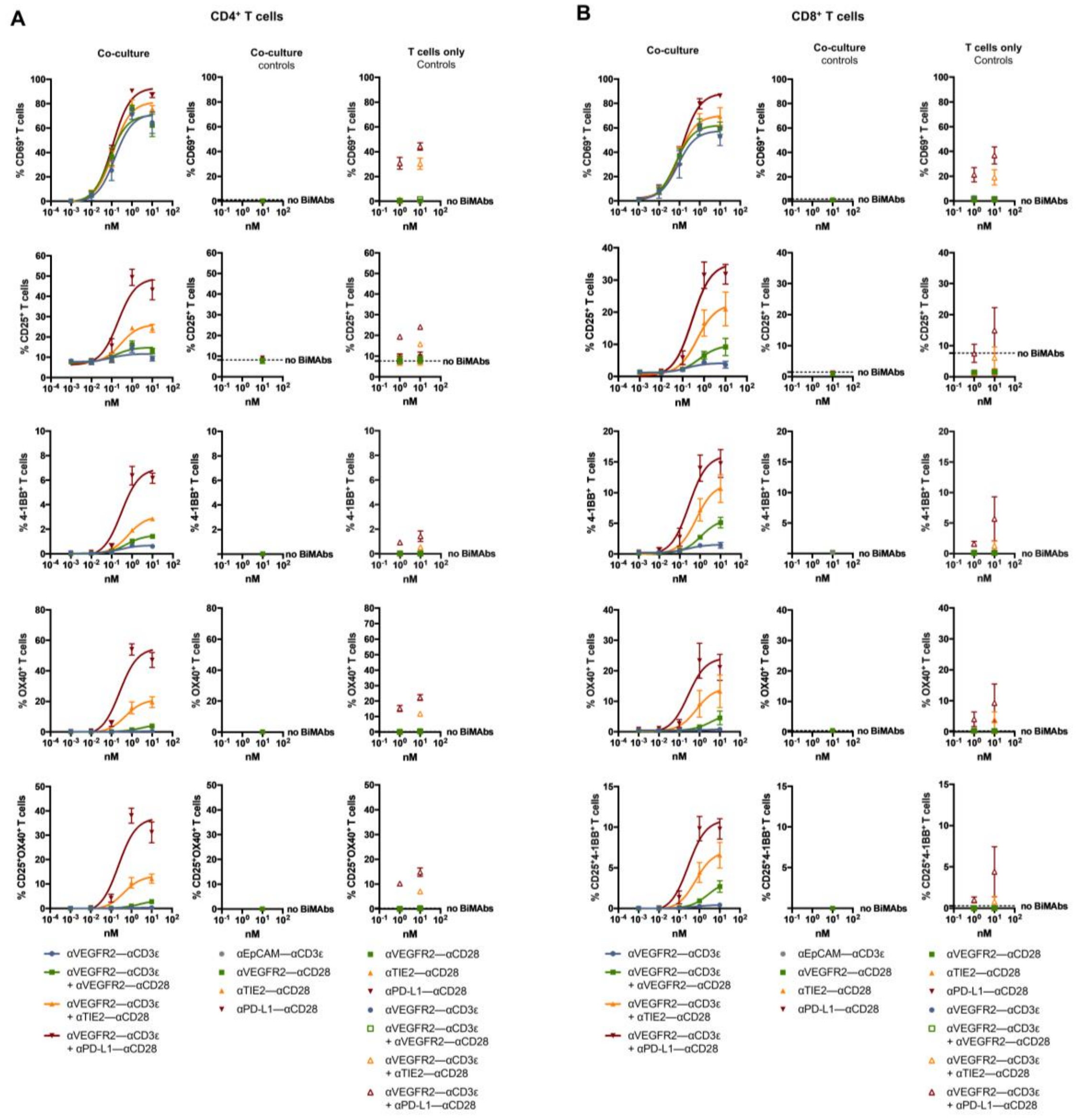

C

EC$_{50}$ values of stimulatory and co-stimulatory bsAb in functional T cell assays

EC$_{50}$ [nM]	αVEGFR2—αCD3ε	αVEGFR2—αCD3ε + αVEGFR2—αCD28	αVEGFR2—αCD3ε + αTIE2—αCD28	αVEGFR2—αCD3ε + αPD-L1—αCD28
CD4+ T cell Activation (CD69+)	0.14	0.08	0.12	0.11
CD4+ T cell Activation (CD25+OX40+)	0.94	2.58	0.42	0.23
CD8+ T cell Activation (CD69+)	0.08	0.07	0.09	0.14
CD8+ T cell Activation (CD25+4-1BB+)	0.63	2.64	0.65	0.28

EC$_{50}$ values in nM were calculated with GraphPad Prism™ Software using non-linear regression log (agonist) vs. response variable slope with a robust fit.

Supplementary Figure S1. Titration of recombinant bispecific antibodies to achieve optimal T cell activation. Freshly isolated T cells were kept without ("T cells only") or in coculture with HUVEC in the presence of purified bsAb, αVEGFR2–αCD3ε and/or αVEGFR2–αCD28 and/or αTIE2–αCD28 and/or αPD-L1–αCD28, for 24 hours at different concentrations (0.001-10 nM). The carcinoma-reactive bsAb αEpCAM–αCD3ε was also used in control cocultures. Cells were collected and evaluated by flow cytometry for expression of activation markers CD69, CD25, 4-1BB and OX40 by CD4$^+$ T cells (**A**) and CD8$^+$ T cells (**B**). Data are presented as

mean values of marker-positive cells ± SEM from 3 independent experiments. (**C**) Table showing EC_{50} values of bsAb titrations regarding the induction of T cell activation markers (CD69, CD25/OX40, CD25/4-1BB) in HUVEC-T cell cocultures shown in (**A**) and (**B**). EC_{50} values in nM were calculated with GraphPad Prism™ Software using non-linear regression log (agonist) vs. response variable slope with a robust fit.

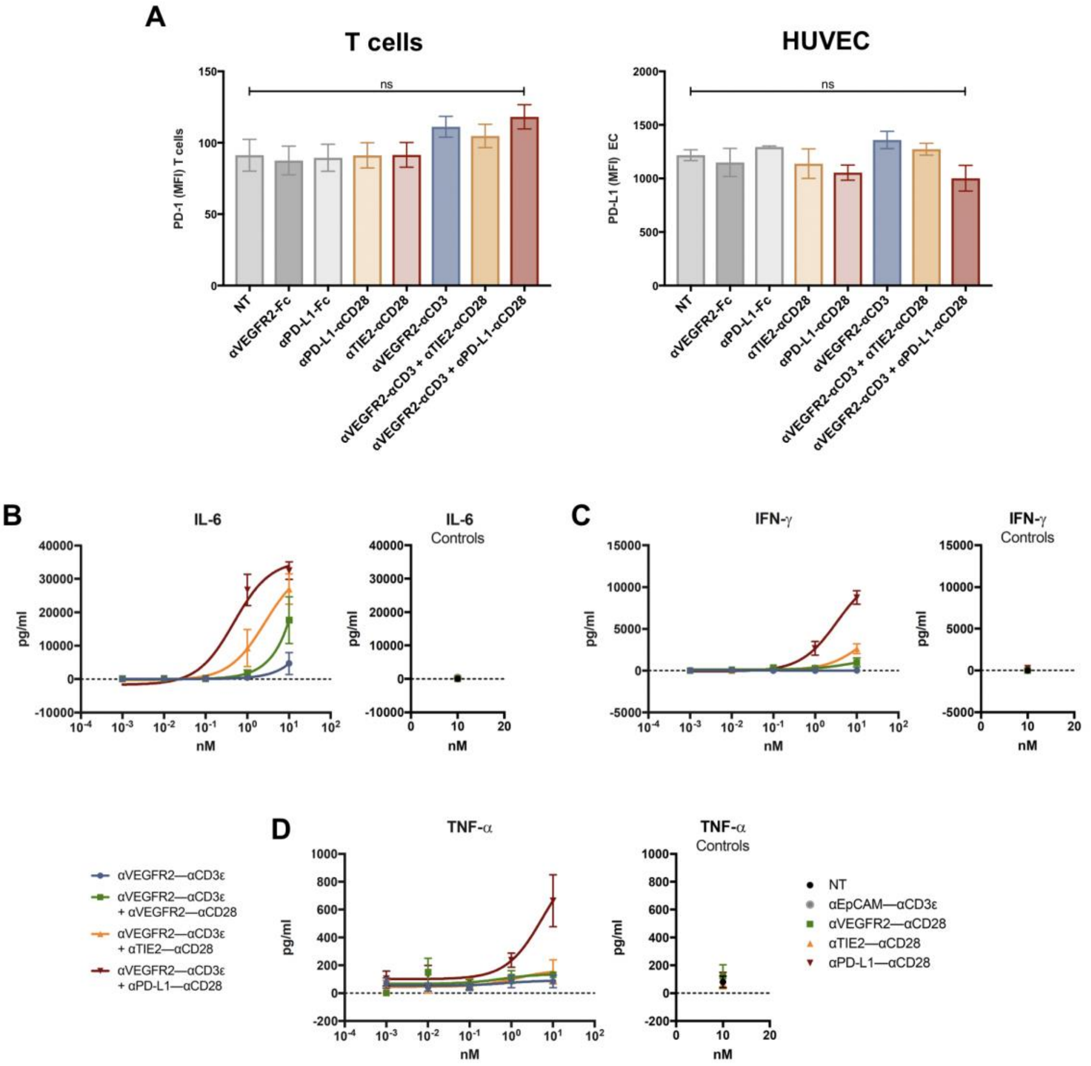

Supplementary Figure S2. (**A**) Analysis of PD-1 expression on $CD3^+$ T cells and PD-L1 expression on HUVEC after an HUVEC–T cell 24h coculture in the presence of the indicated EC/T cell-reactive bsAb or VEGFR2-Fc for control. (**B**–**D**) Quantification of cytokine secretion after EC-T cell 24h coculture in the presence of EC/T cell-reactive bsAb. T cells were cocultured with HUVEC overnight in the presence of *α*VEGFR2–*α*CD3ε alone or in combination with *α*VEGFR2–*α*CD28, *α*TIE2–*α*CD28 and *α*PD-L1–*α*CD28 for 24 hours at different concentrations (0.001-10 nM). For control, costimulatory bsAb were used alone at 10 nM. Supernatants were collected and analyzed by ELISA for secreted amounts of IL-6 (B), IFN-*γ* (C) and TNF-*α* (D) (pg/ml). Data are presented as mean ± SEM from 3 independent experiments.

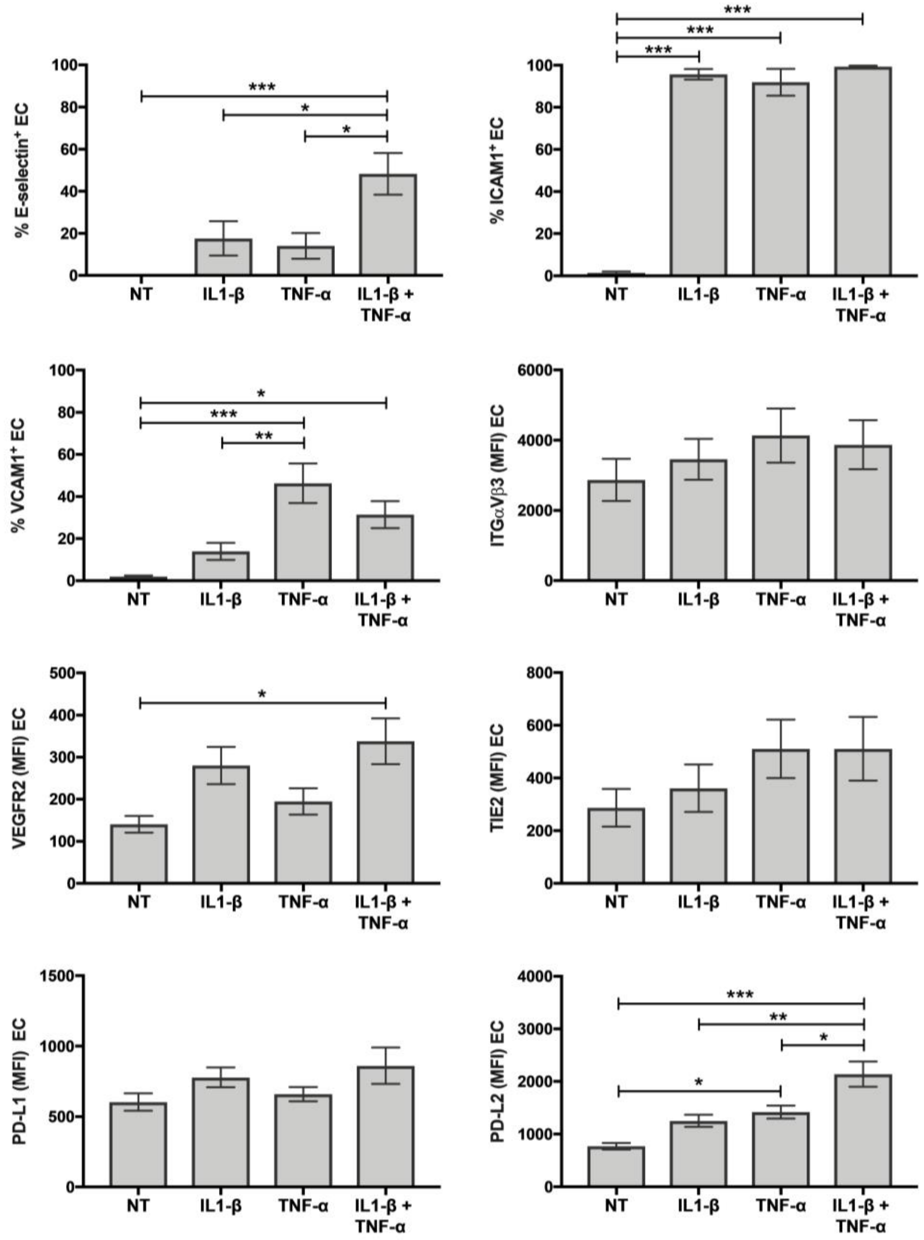

Supplementary Figure S3. Endothelial cell expression profiling after cytokine activation. HUVEC activation was investigated after overnight stimulation with pro-inflammatory cytokines, IL-1β (20 ng/ml) or/and TNF-α (10 ng/ml). Graphs represent the expression levels (MFI or % of positive expressing cells) of EC adhesion molecules involved in lymphocyte adhesion and transmigration (E-selectin, ICAM1, VCAM1, integrin $\alpha_v\beta_3$), endothelial cell growth (VEGFR2, TIE2), as well as T cell immune checkpoint blockade (PD-L1, PD-L2). Data are presented as mean values of % marker-expressing cells or means of MFI values ± SEM from 5 independent experiments. Statistical analysis by one-way ANOVA test followed by Tukey's multiple comparison test; *$p < 0.05$; **$p < 0.01$; ***$p < 0.001$.

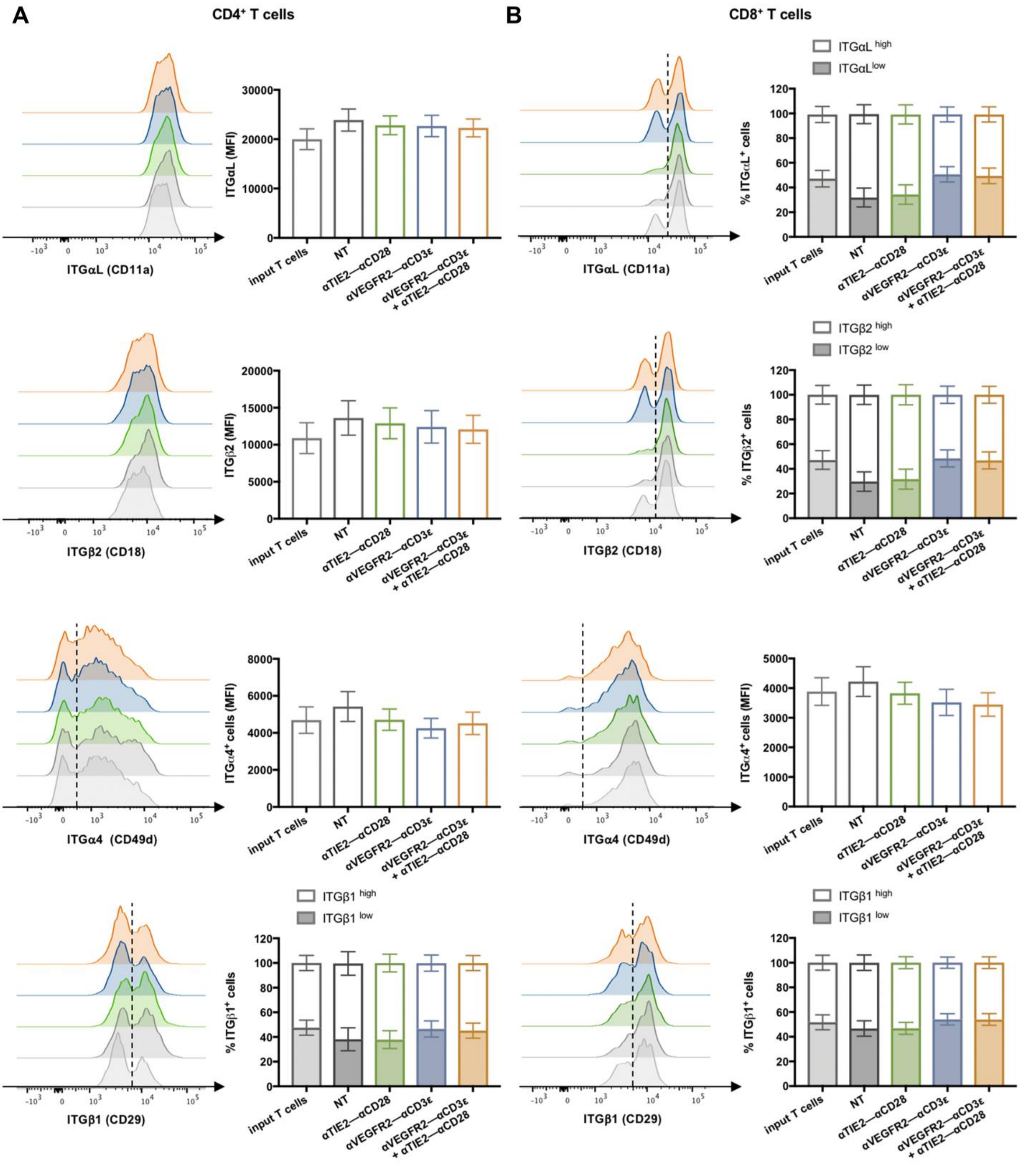

Supplementary Figure S4. Integrin expression on T cells after bsAb stimulation for 4 h. T cells were cocultured with HUVEC for 4 h in the presence of (co)stimulatory bsAbs, αVEGFR2–αCD3ε and/or αTIE2–αCD28 or left untreated. For comparison, T cells cells were analyzed before coculture ("input T cells"). Expression of integrins α_L (CD11a), β_2 (CD18), α_4 (CD49d) and β_1 (CD29) by CD4⁺ (**A**) and CD8⁺ (**B**) T cells were analyzed using flow cytometry. Data are presented as mean values ± SEM from 6 independent experiments.

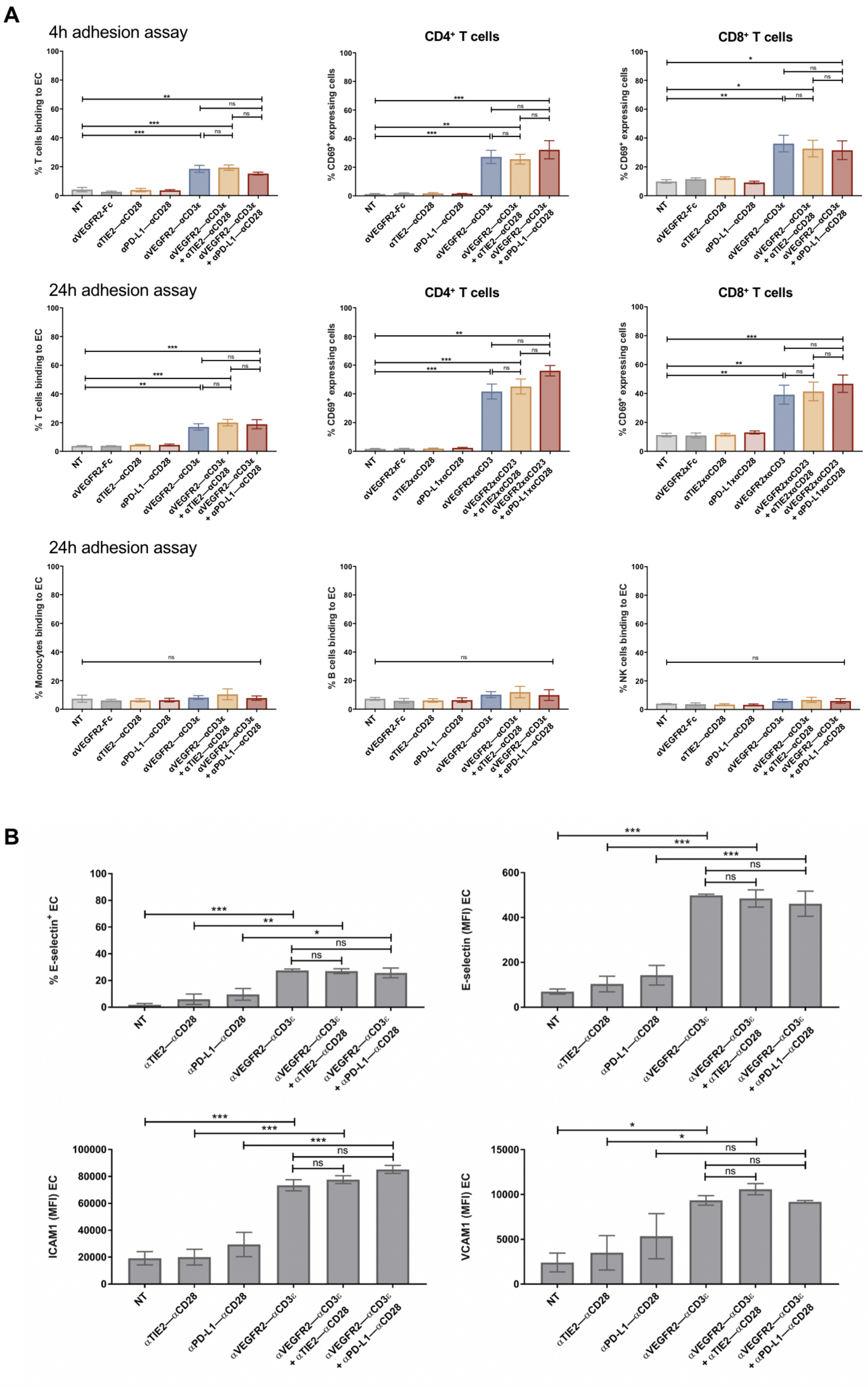

Supplementary Figure S5. (**A**) Adhesion assay with unseparated PBMC and HUVEC. Proportions of T cells, monocytes, B cells and NK cells in 3 independent preparations of PBMC from healthy donors were 64%, 6%, 4% and 3% respectively. Upper row:

Percentages of CD3+ T cells binding to HUVEC and activation of CD4+ and CD8+ T cells after 4 h in the presence (co)stimulatory bsAbs, αVEGFR2–αCD3ε and/or αTIE2–αCD28 or αPD-L1–αCD28, VEGFR2-Fc for control, or not treated (NT). Middle row: Percentages of CD3+ T cells binding to HUVEC and activation of CD4+ and CD8+ T cells after 24 h in the presence (co)stimulatory bsAbs, αVEGFR2–αCD3ε and/or αTIE2–αCD28 or αPD-L1–αCD28, αVEGFR2-Fc for control, or not treated (NT) Lower row: Adhesion of CD14+ monocytes, CD19+ B cells and NKp46+ NK cells, respectively, after 24 h of coculture in the presence of the indicated bsAb. (**B**) Upregulation of endothelial cell adhesion molecules in the presence of tumor-cell conditioned medium. HUVEC–T cell cocultures were conducted for 24 h of in the presence of the indicated (co)stimulatory bsAb (1 nM) in RPMI-1640 medium conditioned by growing MCF-7 tumor cells. EC were dissociated from plates and expression of adhesion molecules E-selectin, ICAM1 and VCAM1 was evaluated by flow cytometry. Statistical analysis of 3 independent experiments was conducted by one-way ANOVA followed by Tukey's multiple comparison test; ns, not significant; *$p < 0.05$; **$p < 0.01$; ***$p < 0.001$.

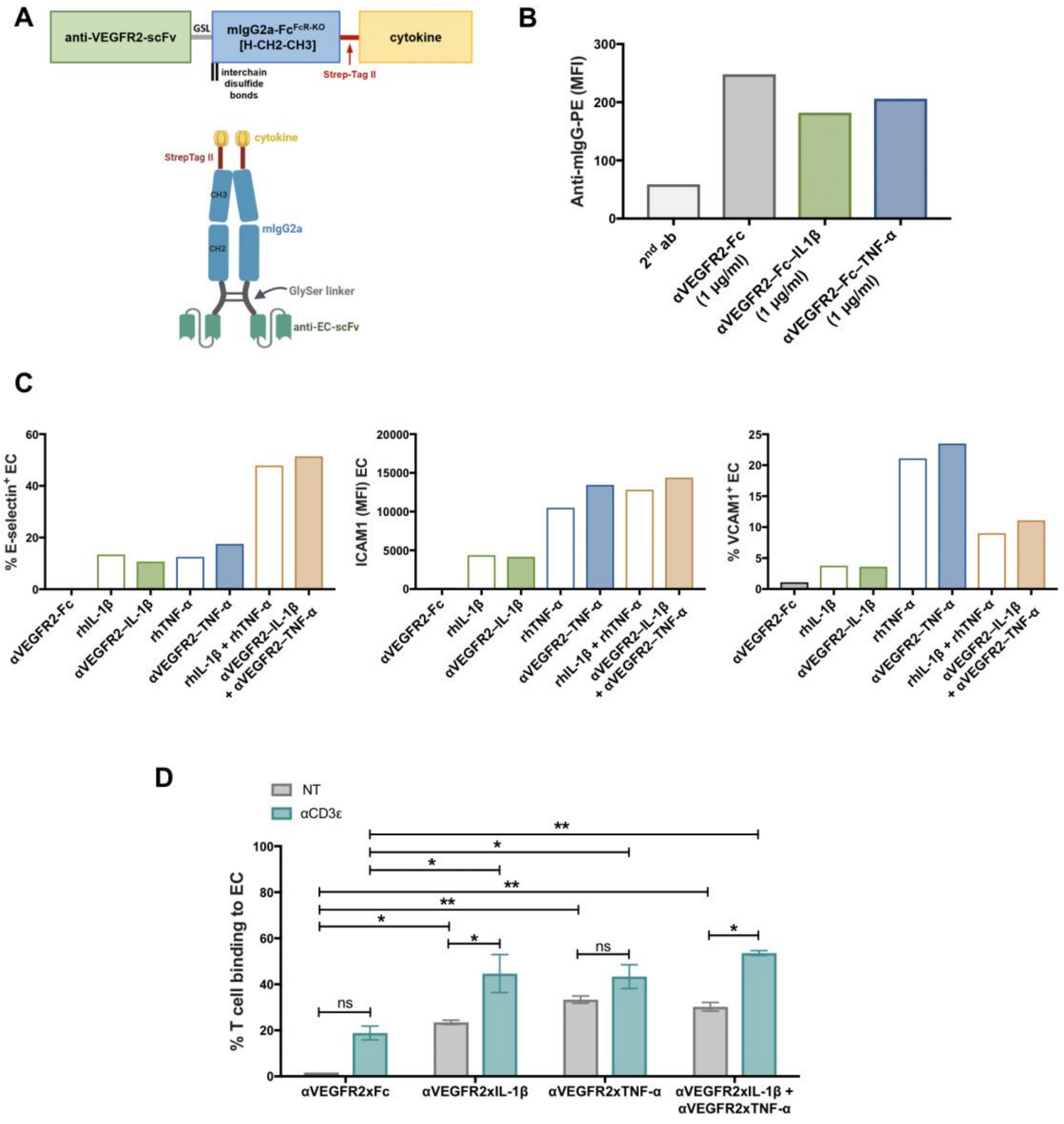

Supplementary Figure S6. Anti-VEGFR2-Fc-cytokine fusion proteins increase T cell adhesion. (**A**) Schematic drawing of αVEGFR2-mIgG2a-Fc-hTNF-α/hIL-1β fusion proteins. (**B**) Cytokine fusion proteins and αVEGFR2-mIgG2a bind to untreated HUVEC as analyzed by flow cytometry with using goat anti-mouse Ig-PE. (**C**) Expression of E-selectin, ICAM1 and VCAM1 by HUVEC cultured with αVEGFR2-cytokine fusion proteins or recombinant human IL-1β (20 ng/ml) or TNF-α (10 ng/ml) for 24 h. (**D**) Firm T cell

adhesion to HUVEC in the presence of αVEGFR2-mIgG2a-Fc-IL-1β or αVEGFR2-mIgG2a-Fc-TNF-α fusion proteins, or a combination of both. After an overnight coculture and extensive washing, bound T cells were harvested and quantified using precision counting beads. Shown are mean values ± SEM of bound T cells as percentages of input T cells from 3 independent experiments. Statistical analysis by two-way ANOVA test followed by Tukey's multiple comparison test; ns, not significant; $^*p < 0.05$; $^{**}p < 0.01$.

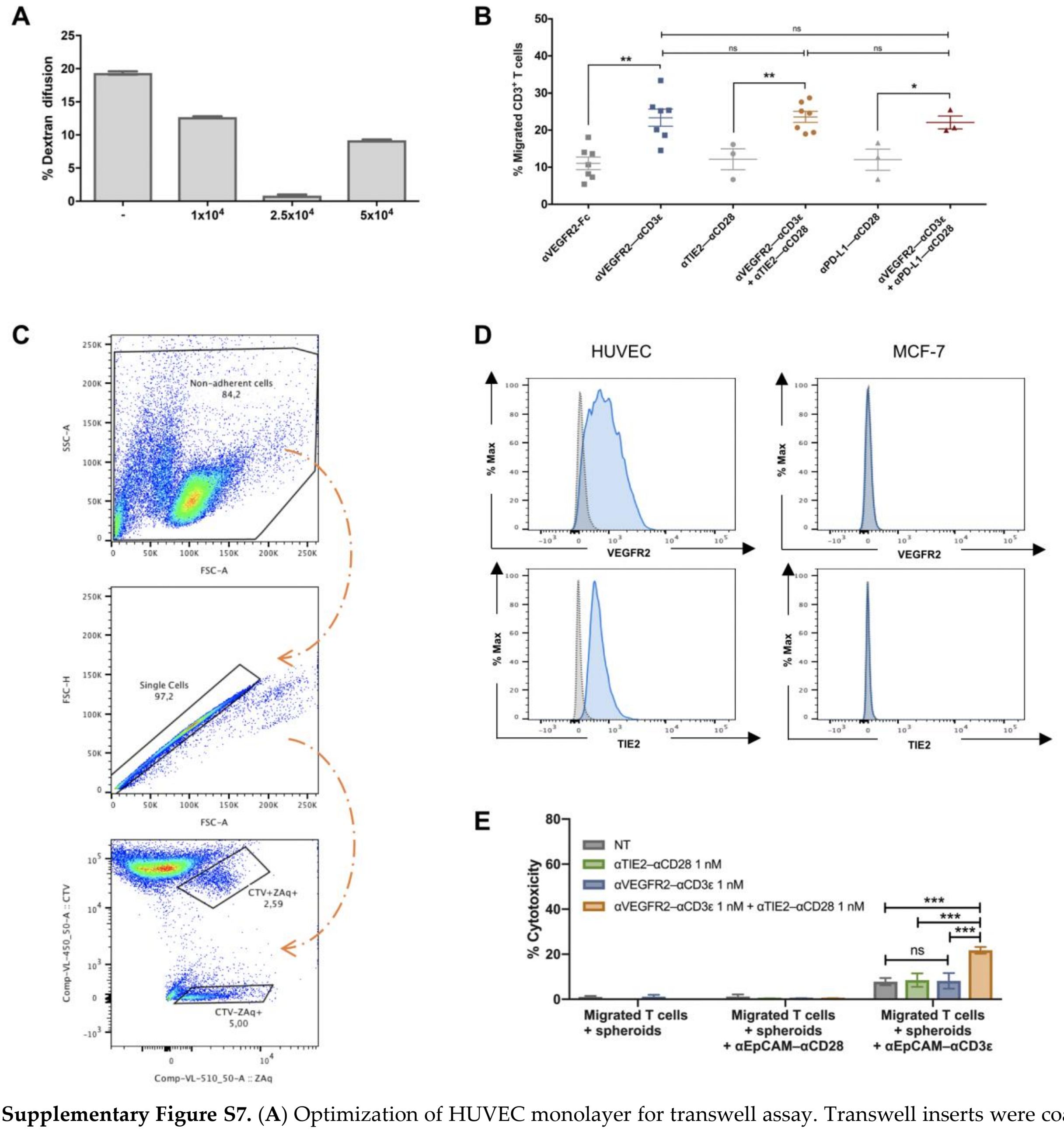

Supplementary Figure S7. (**A**) Optimization of HUVEC monolayer for transwell assay. Transwell inserts were coated with 1% human fibronectin at 15 μg/cm². HUVEC were seeded on coated inserts at different densities ($1x10^4$, $2.5x10^4$ and $5x10^4$) and left for 48 h until a confluent monolayer was achieved. HUVEC monolayer permeability was evaluated by the presence of FITC-dextran (3-5 kDa) in the supernatant from the lower chamber using a fluorescence reader. Data is presented as mean ± SEM of technical triplicates. (**B**) T cell migration is increased by αVEGFR2–αCD3ε treatment alone or together with αTIE2–αCD28/αPD-L1–αCD28 costimulation. $2.5x10^4$ HUVEC were seeded onto fibronectin-coated transwell inserts and left for 48 h until a confluent monolayer was obtained. Purified $CD3^+$ T cells ($1x10^6$) were added to the insert together with αVEGFR2–αCD3ε (0.5 nM), αTIE2–αCD28 (1 nM), αPD-L1–αCD28 (1 nM), or αVEGFR2-Fc for control. After 24 h, migrated $CD3^+$ T cells were collected from the lower chamber and quantified by flow cytometry using counting beads. The figure indicates the percentage of transmigrated $CD3^+$ T cells from input $CD3^+$ T cells

added to the upper well. Data is presented as mean ± SEM from 3-9 independent experiments. (**C**) Flow cytometry gating scheme corresponding to Figure 5D that analyzes dead (ZAq^{+}) cells in the non-adherent fraction of cells present in the transwell insert after a 24h transmigration assay. CellTrace Violet (CTV) is used to label T cells while detached ZAq^{+} HUVEC are CTV^{-}. (**D**) Flow cytometric analysis of MCF-7 cells in comparison to HUVEC for cell surface expression of VEGFR2 and TIE2 using fluorochrome-labeled antibodies. (**E**) Cytotoxicity against MCF-7 tumor spheroids by normalized numbers of migrated T cells. Fibronectin-coated transwell inserts were incubated with $2.5x10^{4}$ HUVEC and left for 48 h until reaching a confluent monolayer. Purified $CD3^{+}$ T cells ($0.5x10^{6}$ cells) were added to the upper chamber together with αVEGFR2–αCD3ε ± αTIE2–αCD28 (1 nM) or without bsAb. MCF-7 spheroids were in a separated plate together with αEpCAM–αCD3ε (10 nM) or αEpCAM–αCD28 (10 nM). After 24 h of migration, the inserts were removed and migrated T cells were counted by flow cytometry using precision counting beads. T cell numbers were normalized to T cells migrated in the absence of bsAb and transferred onto MCF-7 spheroids for additional 48 h. The cytotoxic capacity of T cells was evaluated by measuring LDH release to the supernatant. Data are presented as mean values of % maximal LDH release ± SEM from 3 independent experiments. Statistical analysis by one-way ANOVA (B) or two-way ANOVA (C) followed by Tukey's multiple comparison test; ns, not significant; *$p < 0.05$; **$p < 0.01$; ***$p < 0.001$.
